# Genome‐wide evolutionary signatures of climate adaptation in spotted sea bass inhabiting different latitudinal regions

**DOI:** 10.1111/eva.13551

**Published:** 2023-04-27

**Authors:** Baohua Chen, Zhixiong Zhou, Yue Shi, Jie Gong, Chengyu Li, Tao Zhou, Yun Li, Dianchang Zhang, Peng Xu

**Affiliations:** ^1^ State Key Laboratory of Mariculture Breeding, College of Ocean and Earth Sciences Xiamen University Xiamen China; ^2^ Shenzhen Research Institute of Xiamen University Shenzhen China; ^3^ Fujian Key Laboratory of Genetics and Breeding of Marine Organisms, College of Ocean and Earth Sciences Xiamen University Xiamen China; ^4^ The Key Laboratory of Mariculture, Ministry of Education Ocean University of China Qingdao China; ^5^ South China Sea Fisheries Research Institute Chinese Academy of Fishery Sciences Guangzhou China

**Keywords:** climate adaptation, evolutionary signatures, population structure, spotted sea bass

## Abstract

Consideration of the thermal adaptation of species is essential in both evolutionary biology and climate‐change biology because it frequently leads to latitudinal gradients of various phenotypes among populations. The spotted sea bass (*Lateolabrax maculatus*) has a broad latitudinal distribution range along the marginal seas of the Northwest Pacific and thus provides an excellent teleost model for population genetic and climate adaptation studies. We generated over 8.57 million SNP loci using whole‐genome resequencing from 100 samples collected at 14 geographic sites (five or ten samples per site). We estimated the genetic structure of the sampled fish and clustered them into three highly differentiated populations. The genetic differentiation pattern estimated by multivariable models combining geographic distance and sea surface temperature differences suggests that isolation by distance and isolation by environment both have significant effects on this species. Further investigation of genome‐wide evolutionary signatures of climate adaptation identified many genes related to growth, muscle contraction, and vision that are under positive natural selection. Moreover, the contrasting patterns of natural selection in high‐latitude and low‐latitude populations prompted different strategies of trade‐offs between growth rate and other traits that may play an essential role in adaptation to different local climates. Our results offer an opportunity to better understand the genetic basis of the phenotypic variation in eurythermal fishes inhabiting different climatic regions.

## 1 INTRODUCTION

Water temperature is one of the most critical climatic factors for fishes, determining various physiologic phenotypes and strongly affecting their fitness and distribution (Payne et al., 2016). The natural population structure of fishes is also sensitive to water temperature due to adaptation to local climates (Sandstrom et al., 1995). Many studies have suggested that fishes living in different temperature environments undergo spatially heterogeneous natural selection (Bradbury et al., 2010; Hasselman et al., 2013; Lannig et al., 2003). Consequently, populations inhabiting different latitudes may undergo inheritable phenotypic differentiation in response to selective pressures (Kawecki & Ebert, 2004; Leimu & Fischer, 2008).

Many ecological studies have revealed that teleosts that are distributed at high latitudes tend to have higher growth potential (i.e., a higher maximum growth rate) than their low‐latitude conspecies (Conover et al., 1997; Imsland et al., 2000; Lindgren & Laurila, 2005). High growth potential is usually accompanied by reductions in resistance to predation (Lankford et al., 2001), swimming performance (Billerbeck et al., 2001), and metabolic rate (Sylvestre et al., 2007), which can be described as different trade‐off strategies in resource allocation (Steiner & Pfeiffer, 2007). Indeed, adaptation to different temperatures involves shifts in a broad range of growth‐related trade‐offs. For example, Roze et al. (Roze et al., 2013) identified tightly interrelated trade‐offs among thermal growth sensitivity, hypoxia tolerance, and growth in rainbow trout (*Oncorhynchus mykiss*). Matte et al. (Matte et al., 2020) studied the mortality‐to‐growth ratio of neighbouring brook trout (*Salvelinus fontinalis*) populations and revealed that this ratio increased linearly with increasing temperature. This could result in different trade‐offs related to mortality and growth in this species. In addition, it has been widely discussed in recent years that trade‐offs between growth and predator avoidance involving various physiological and behavioural traits such as swimming ability (Watson et al., 2018), lipid allocation (Mogensen & Post, 2012) and surplus aerobic metabolic capacity (Norin & Clark, 2017) play a critical role in teleost climate adaptation (Mogensen & Post, 2012; Norin & Clark, 2017; Watson et al., 2018).

**FIGURE 1:**
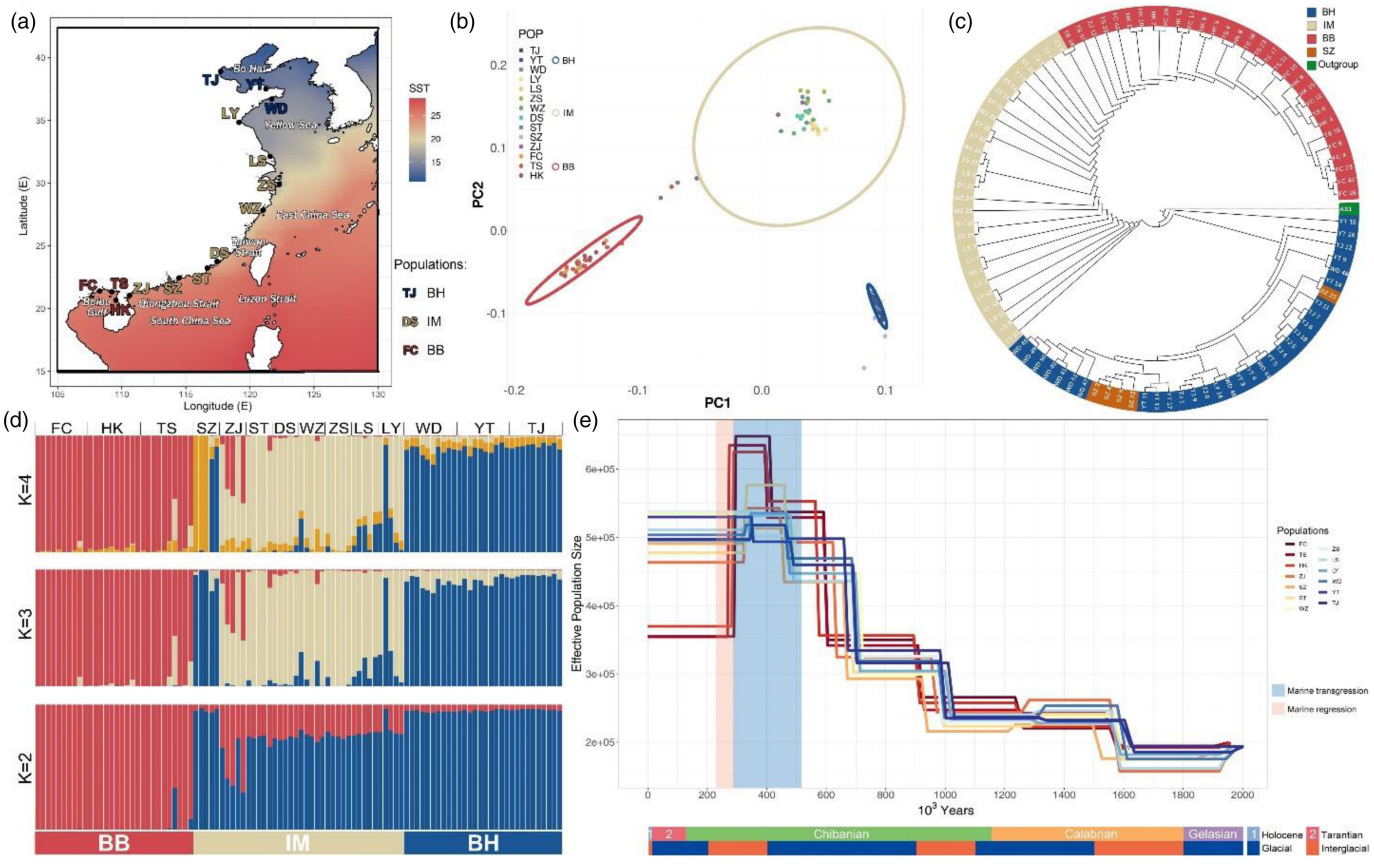
Population genomics and phylogenetic analysis indicating the genetic structure and evolutionary relationship of spotted sea bass (*Lateolabrax maculatus*) populations. (a) Sampling sites along the Chinese coastline. Offshore waters are coloured according to the median annual SST. The ellipses in different colours show the locations of the three main populations of these species. (b) Principal component analysis (PCA) showing genetic distance among samples. The genetic diversity of each population is shown by confidence ellipses (BH, BH, and IM). (c) Neighbor‐joining tree demonstrating the phylogenetic relationship among all samples. The tree is rooted by taking an Asian sea bass as an outgroup. Sampling sites are colored according to the population to which they belong. The sampling site, Shenzhen (SZ), is highlighted in orange because all fishes sampled there might be escaped fishes. (d) Ancestral admixture analysis reveals the genetic structure of all samples. The populations to which each sample belongs and the site from which it was obtained are indicated at the top. (e) Demographic analysis indicates the historical effective population size of *L*. *maculatus* populations. The pale red and blue stripes in the background indicate marine transgression and regression events that may be associated with the expansion of BB populations. The geologic ages are plotted below the x‐axis and are presented in different colours.

The spotted sea bass (*Lateolabrax maculatus*) was recently discriminated from the Japanese sea bass (*L*. *japonicus*) as a newly recharacterized species with noticeable morphological and genetic differences (Yokogawa, 1998). Taking advantage of their eurythermic and euryhaline characteristics, spotted sea bass are distributed from the tropical Beibu Gulf in the South China Sea to the mid‐temperate Bohai Gulf (Figure 1a). The sea surface temperature (SST) in winter can fall to below 2°C in the northern Bohai Gulf and remain as high as 15°C in the Beibu Gulf, while the difference in SST in these two locations is very small in summer. Spotted sea bass inhabiting high‐ and low‐latitude areas show different life‐history strategies, as do many ectothermic animals. The northern populations in the Bohai Gulf have stronger growth potential and motor ability and higher cold resistance than their southern conspecifics in the South China Sea (Hu et al., 2019), making this species a good model through which to define the genetic basis of thermal adaptation. Our previous population genetic study employing double‐digest restriction site‐associated DNA (ddRAD) technology preliminarily investigated the population structure of this species (Zhao et al., 2018). However, the lack of a reference genome made it impossible to obtain a sufficient number of SNP loci to support an in‐depth investigation of the genetic mechanism that underlies thermal adaptive evolution in this species.

In the current work, we used a whole‐genome sequencing (WGS) approach to detect genome‐wide SNP sites based on an assembled reference genome of *L*. *maculates* (Chen et al., 2019) and reinvestigated the population structure in this species. Furthermore, we investigated how geographic and climatic factors drove the genetic differentiation of this species using isolation‐by‐distance (IBD) and isolation‐by‐environment (IBE) approaches and used a combination of high‐resolution selective‐sweep scanning and Bayesian‐based GxE analysis to identify the adaptive evolutionary signatures of spotted sea bass distributed across a wide latitudinal gradient. The results increase our understanding of the genetic differentiation patterns and adaptive genetic mechanisms of eurythermal fish populations under changing climatic conditions.

## 2 MATERIALS AND METHODS

### 2.1 Sampling, sequencing, and SNP calling

We performed whole‐genome resequencing of 100 spotted sea bass collected from 14 sites (ten samples from each site in the Beibu Gulf and the Bohai Gulf and five samples from each other site) along the coastline of China (Figure 1a and Table S1). White muscles were collected from fish anesthetized with eugenol (200 mg/L for 15 min). Muscle samples were lysed in SDS digestion buffer containing proteinase K. DNA extraction was performed using the phenol–chloroform method. For each sample, a 250‐bp insert‐size library was prepared from approximately 0.5 μg of DNA according to the procedures outlined in the Illumina DNA Prep Reference Guide. All libraries were sequenced on the Illumina HiSeq 4000 platform, generating 150‐bp paired‐end reads.

Our previous reference genome assembly was completed with high accuracy and contiguity at the scaffold level (GenBank accession: GCA_004028665.1) (Chen et al., 2019). This version of the genome contains 1639 scaffolds ranging in size from 952 bp to 12.09 Mbp and has an N50 size of 2.79 Mb. However, LACHESIS software, which is now obsolete, was used to process the raw Hi‐C data and chromosome‐level scaffolding, and this may have introduced suboptimal orderings and orientations of the scaffolds (Zhang et al., 2019). Hence, the genome was first refined using a better Hi‐C data processing tool, 3D‐DNA (version 201008) (Dudchenko et al., 2017). The improved genome assembly, which contained 480 scaffolds and had a total length of 589.99 Mbp, had higher integrity than the previous assembly (Figure S1 and Table S1 in Appendix S1). The improved genome also shows higher collinearity with the linkage map of *L*. *maculatus* and the genomes of related species (Figures S2 and S3, and Table S2 in Appendix S1). The quality of the raw sequencing data was assessed and controlled using SolexaQA++ (version 3.1.1) (Cox et al., 2010). SNP calling was then performed using GATK (version 4.0.2.1) (McKenna et al., 2010) under the guidance of GATK Best Practices (van der Auwera et al., 2013). Finally, hard filtering was applied to the raw SNP callset. Detailed information about these steps can be found in Appendix S1.

### 2.2 Population genetics analysis

As highly linked SNPs are redundant for population genetics analysis, the final set was shrunk using PLINK with the parameter “‐‐indep‐pairwise 5 1 0.9” in each population. Three genetic diversity indices, observed heterozygosity (Ho), gene diversity (Hs), and Fis statistics, were calculated using the R package “hierfstat” (version 0.5–7) (Goudet, 2005). The mean *Tajima's D* across all chromosomes was estimated using “VCFtools” (version 0.1.17) (Danecek et al., 2011). In principal component analysis (PCA), the original variant call format (VCF) file was converted to CoreArray Genomic Data Structure (GDS) format using the R package “gdsfmt” (version 1.22.0) (Zheng et al., 2012). Eigenvectors and eigenvalues were then calculated using the R package “SNPRelate” (version 1.16.0) (Zheng et al., 2012) with default parameters. “Frappe” software (version 1.0) (Tang et al., 2005) (version 1.1) was employed to estimate the genetic ancestry of each sample with 100,000 expectation maximization (EM) iterations and up to four ancestral populations. We constructed a neighbour‐joining (NJ) tree of all samples using a command‐line‐based collection of utilities, VCF‐kit (version 0.1.6) (Cook & Andersen, 2017). The size histories of all populations were estimated from whole‐genome sequence data by SMC++ (version 1.15.4) (Terhorst et al., 2017). As molecular clocks have not been calculated in *L*. *maculatus* or its closely related species, a mutation rate of 2.0 × 10‐9 per base per generation calculated in the Atlantic herring (*Clupea harengus*) (Feng et al., 2017) was used in this study. Homozygous regions longer than 5 kb were treated as missing. The polarization error was set to the default value (0.5). All other arguments were set to default values.

### 2.3 Isolation by distance and environment analysis

The pairwise *F*_ST_ values among populations were calculated within each chromosome using Plink2 (version v2.00a3) (Chang et al., 2015) with the “‐‐fst” parameter based on Weir and Cockerham's method (Weir & Cockerham, 1984). Genetic distance was calculated as *F*_ST_/(1 − *F*_ST_) (Rousset, 1997). Three geographic distances (great‐circle distance, *D*_gcc_; distance along the coastline, *D*_csl_; and distance along latitude, *D*_lat_) and three temperature differences (annual maximum, minimum, and median sea surface temperature differences, denoted SST_max_, SST_min_, and SST_min_, respectively) were used to model the genetic differentiation. *D*_gcc_ was calculated from longitude and latitude coordinates using the following equations:

$$D_{\mathrm{gcc}}=2R\cdot \arcsin\left(\sqrt{\sin^{2}\left(\frac{\varphi_{2}-\varphi_{1}}{2}\right)+\cos\left(\varphi_{1}\right)\cos\left(\varphi_{2}\right)\sin^{2}\left(\frac{∆\lambda }{2}\right)}\right),$$

where *R*, *φ1*, *φ2*, and *∆λ* are the Earth's radius (6371.393 km), the latitude of one site, the latitude of the other site, and the difference in longitude between the two sites, respectively. *D*_csl_ was calculated based on the preprocessed OpenStreetMap data obtained from the Planet OSM website (https://osmdata.openstreetmap.de). Raw data in shapefile format were transformed into “Mercator” projection using the R package “sf” (version 0.9.7) and then divided into 0.1 × 0.1‐km^2^ grids. Finally, *D*_csl_ was calculated as the sum length of the diagonal lines of all grids connecting two places. Daily SSTs from 1971 to 2010 were extracted from NOAA high‐resolution SST data (https://psl.noaa.gov/) (Reynolds et al., 2007). The “netCDF4” Python library (version 1.5.5.1) was used to parse the NetCDF4 file. We averaged SST values within a 0.25‐degree grid covering each site.

To model how a single factor influences the genetic distance, single‐factor linear regression between genetic distances and exogenous geographic and climatic variables was implemented using the “statsmodels” Python library (version 0.12.1) for IBD and IBE analyses. When attempting to dissect the effects of IBD and IBE patterns, the main challenge we faced was multicollinearity among exogenous variables. To address this, three mutually independent methods with high tolerances for multicollinearity were employed to solve the problem. Regularized least squares (RLS) regression is a family of relatively new linear regression techniques that were recently introduced for solving problems of confounding and causality plaguing genome‐environment association studies (Frichot & Francois, 2015). The generalized dissimilarity model (GDM) is a matrix regression technique that is powerful when applied to the model genetic distance between sampling sites against geographic and environmental variables (DeSilva & Dodd, 2020; Geue et al., 2016). The third method, canonical correlation analysis (CCA), is a powerful and widely used method for coping with multicollinearity in IBD and IBE analysis (Prunier et al., 2015). We conducted regularized least‐squares regression using the “linear_model” module contained in the “sklearn” python library (version 0.24.0). In RLS regression, unimportant variables were first discarded by least absolute shrinkage and selection operator (LASSO) regression. The remaining variables were then used to build an elastic net model with an alpha value of 0.5. Both LASSO and elastic net models were built using the “sklearn” python library (version 0.24.0). The “gdm” R package (version 1.4.0) (Mokany et al., 2022) was employed to fit a GDM. The model significance and variable importance were estimated using the “varImp” function with 5000 matrix permutations. The “isplineExtract” function in the “gdm” R package was used to extract an I‐spline whose maximum height indicates the significance of predictors (Ferrier et al., 2007) for each predictor from the resulting GDM objects. In CCA, all descriptive statistics were calculated using the “calc.yhat” function in the “yhat” R package (version 2.0–3) (https://cran.uib.no/web/packages/yhat/index.html). Their confidence intervals were generated after 5000 bootstraps by the “booteval.yhat” function.

### 2.4 Identification of temperature‐associated SNP loci and genes

Temperature‐associated SNP loci were identified based on SST_max_ and SST_min_ using Baypass (version 2.2) (Gautier, 2015). The population covariance matrix was first estimated in the core model with default parameters and was then used as an extra input in standard covariate mode to estimate the Bayes factor (BF) of each SNP with default parameters. Bayes factors were estimated in deciban (dB) units. Only SNPs with a decisive evidence level (BF > 20 dB) of associations were identified as SST_max_‐ or SST_min_‐associated SNP loci (Hoijtink et al., 2019). RDA was performed using the “vegan” (version 2.6–4) library in R (Dixon, 2003). The RDA model was first built using the “rda” function with default parameters. The *p* values of SNPs were then calculated from their loading scores with a chi‐square distribution. The overlaps between outliers identified using BayPass and vegan were considered temperature‐associated SNPs. Finally, Metascape (version 3.5) (Zhou et al., 2019) was employed for enrichment analysis based on genes located around these SNPs.

### 2.5 Selective signature detection

A sliding window (window size = 50 kbp and step size = 10 kbp) was used to identify genomic regions that have undergone positive selection in the northern (BH) and southern (BB) populations. The fixation index (*F*_ST_) and nucleotide diversity were estimated using VCFtools (version 0.1.15) (Danecek et al., 2011) for each window. This software was also employed to calculate nucleotide diversity values. The ratio of nucleotide diversity in the BH population to that in the BB population was calculated and then log‐2 transformed (log_2_ *π*_*BH*_ */π*_*BB*_, denoted *ω*). The extent of haplotype homozygosity (*Rsb*) was calculated for each SNP using the R package “rehh” (version 3.0.1) (Gautier et al., 2017) and then averaged within sliding windows. Positively selected regions (PSRs) were identified as regions having absolute *F*_ST_, *ω* and *Rsb* in the 95th percentiles. Genes whose gene bodies overlapped with any PSR were identified as positively selected genes (PSGs). In addition, Tajima's *D* index was estimated using VCF‐kit (version 0.1.6) (Cook & Andersen, 2017) with the parameter “tajima”. The composite selection score (*CSS*) (Avalos et al., 2017) was calculated from *F*_ST_, *ω*, and *Rsb* using an in‐house Python script. Z‐transformed fractional ranks were averaged across the three statistics for each sliding window. The averaged Z scores were then compared against a normal distribution to derive a *p* value. One‐way ANOVA was employed to assess the significance of differences in *F*_ST_, *ω*, *Rsb*, *CSS*, and *Tajima's D* between the BH and BB PSRs.

Finally, Metascape (version 3.5) (Zhou et al., 2019) was used to analyze differential enrichment of Gene Ontology biological processes, Kyoto Encyclopedia of Genes and Genomes (KEGG) pathways, and Reactome gene sets for BH and BB PSGs. Enriched functional categories were subsequently grouped into several supercategories according to the overlaps of genes they harboured. Each supercategory was represented by the most significant category it contained (both name and *p* values).

### 2.6 Prediction of the tertiary structure and thermostability of myosin heavy chain (*myhz2*)

To understand the functional impact of the identified mutations in the myosin heavy chain (*myhz2*) gene on protein thermostability, the dominant alleles of each population and each nonsynonymous mutation were first applied to the reference *myhz2* coding sequence to derive BH‐ and BB‐specific protein sequences. The tertiary structures assumed by proteins with these two sequences were then predicted using I‐TASSER (version 5.1) (Yang et al., 2015) with an identity cut‐off of 0.3. Two approaches were employed to estimate the thermostability of the two structures. The “SCooP” web server (version 1.0) (Pucci et al., 2017) was used to predict the Gibbs–Helmholtz equation associated with the folding transition. In addition, another web server, deepDDG (Cao et al., 2019), was used to predict the changes in protein stability brought about by point mutations using neural networks.

## 3 RESULTS

### 3.1 Sequencing and SNP detection

High‐throughput sequencing generated 3.97 billion pairs of raw reads with a total length of 1.19 Tbp and an average depth of 19.64×. After quality control, 98.42% of the reads (1.17 Tbp) remained for processing in the downstream steps. Of these, 783.6 billion clean reads were mapped to the reference genome of *L*. *maculatus* at mapping ratios ranging from 97.2% to 99.1%. We obtained a total of 8.57 million SNPs from the 100 sampled fish (Figure 1a, Figure S1, and Tables S1‐S3). We also generated a pruned SNP set after the removal of 1.19 million (13.88%) highly linked SNPs. This pruned set was used only in the genetic structure analysis.

### 3.2 Genetic structure of the spotted sea bass

As shown in Figure 1b, the first two principal components in PCA, which explain 3.28% and 1.35% of the variance, respectively, group all samples into three populations. The BH and BB populations include samples collected from the Bohai Gulf and the Beibu Gulf, respectively. All other samples except the Shenzhen (SZ) samples belong to an intermediate population (IM). The 95% confidence ellipses of the three populations do not overlap with each other, implying that they are highly differentiated. Phylogenetic trees support the same division of these samples (Figure 1c and Figure S2). Ancestry proportion estimation reconfirmed that the samples can be divided into three populations when K is set to three. When K is set to two, the IM populations are depicted as an admixture of the BH and BB populations. Most of the samples in the IM population, with the exception of the ZJ samples, were found to harbor more genetic material introgressed from the BH population than from the BB population, (Figure 1d).

We built an NJ tree by taking a related species (the Asian sea bass, AS) as the outgroup (Figure 1c). The rooted phylogenetic tree showed that the spotted sea bass found in Chinese coastal seas originated in the Bohai Gulf and then spread to southern areas. In addition, the historical effective population sizes suggest that during this process the BB populations experienced rapid expansion 410,000 years ago followed by a bottleneck occurring approximately 290,000 years ago (Figure 1e).

### 3.3 Isolation by distance and environment analysis

**FIGURE 2:**
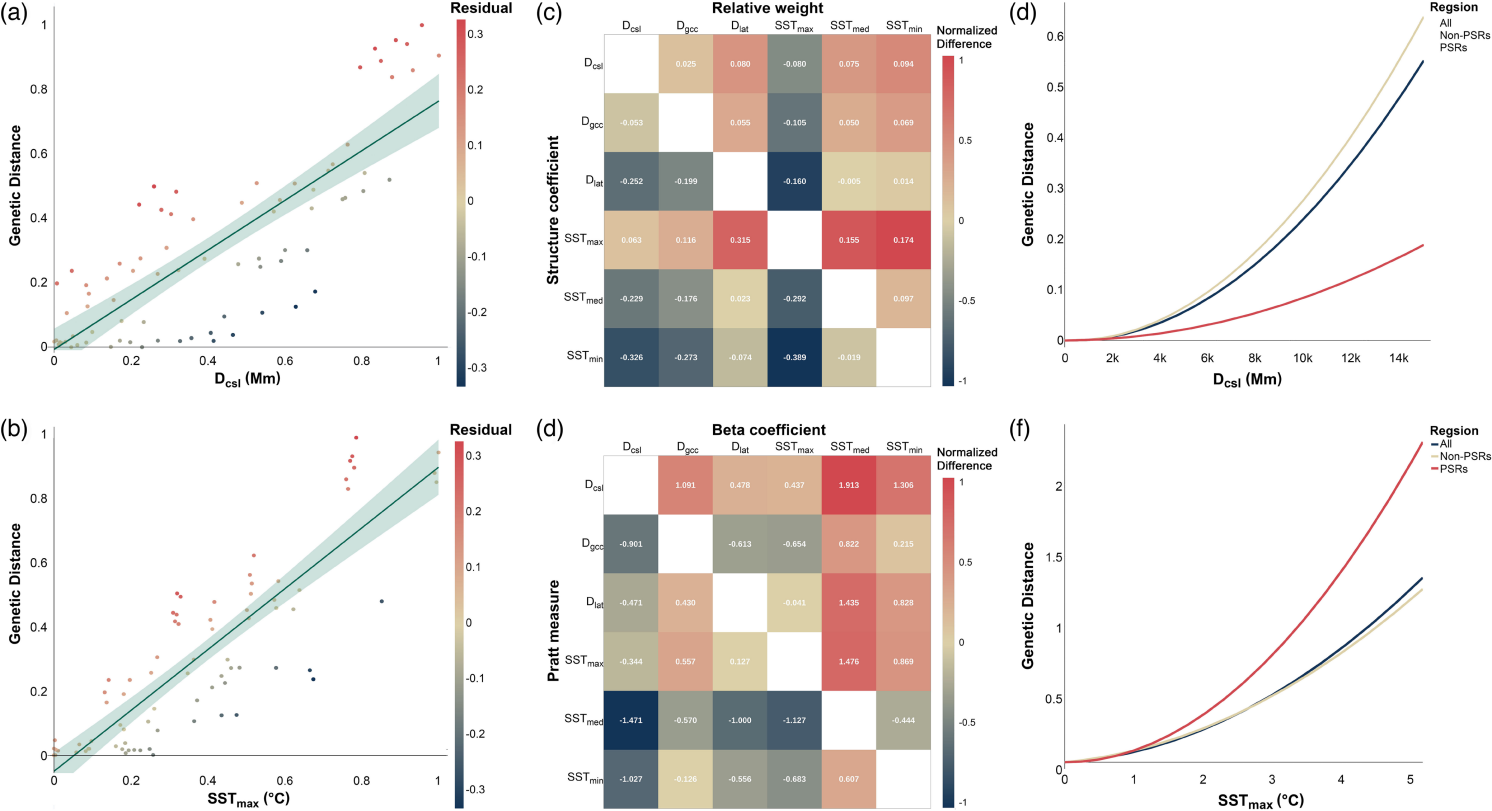
Genetic isolation by distance and environment. (a) OLS regression showing the high linear correlation between the genetic distance and the geographic distance along coastlines (*D*_csl_). The color of the dots indicates the deviation of the predicted value from the observed value. (b) OLS regression showing the high linear correlation between the genetic distance and the difference in annual maximum sea surface temperature (SST_max_). The color of the dots indicates the deviation of the predicted value from the observed value. (c) A final generalized dissimilarity model was built with *D*_csl_ and SST_max_ after removal of other factors of lesser importance. The color of the dots indicates the deviation of the predicted value from the observed value. (d) Pairwise differences of squared structure coefficients (top‐left) and relative weights (bottom‐right) among all exogenous factors. Significant differences between pairs of predictors are indicated by being filled with continuous colours according to their min‐max scaled values. Positive values indicate that the predictor in the line is more critical than the predictor in the column. (e, f) I‐splines indicating how the genetic distance of nonneutral regions (red line), neutral regions (blue line), and the whole genome (khaki line) increases along the *D*_csl_ (e) and SST_max_ (f) gradients.

In the IBD analysis, models fitted using *D*_csl_ and *D*_gcc_ had higher goodness of fit ($R_{\mathrm{adj}}^{2}$ = 0.652 and 0.607, *AIC* = −540.943 and −531.416, for *D*_csl_ and *D*_gcc_, respectively) and greater significance (*p* = 2.57E‐19 and 2.76E‐17) than those fitted using *D*_lat_ ($R_{\mathrm{adj}}^{2}$ = 0.444, AIC = −504.385, and *p* = 1.67E‐11) (Figure 2a, Table 1, and Figure S3). In IBE analysis, SST_max_ showed the sole negative Akaike information criterion (−32.899) and a *p* value (7.53E‐16) that was much lower than those associated with the other two factors, indicating a more significant correlation with genetic distance (Figure 2b, Table 1, and Figure S4).

**TABLE 1:** Linear regression showing the variance in genetic differences explained by differences in geological and environmental factors between populations. The columns in this table show the geographic/climatic factors used to build the models, the coefficients of determination (*R* ^2^), adjusted *R* ^2^, *p* values, log_10_‐transformed likelihoods, Akaike information criteria (AIC), and Bayesian information criteria (BIC) associated with the linear regression models.

Factor	*R* ^2^	$R_{\mathrm{adj}}^{2}$	*p* value	Log‐likelihood	AIC	BIC
IBD						
*D*_csl_	0.544	0.538	1.40E‐14	17.836	−31.673	−26.959
*D*_lat_	0.278	0.268	7.22E‐07	−0.067	4.134	8.847
*D*_gcc_	0.501	0.494	4.31E‐13	14.357	−24.713	−20.000
IBE						
SST_max_	0.577	0.571	7.53E‐16	20.806	−37.612	−32.899
SST_med_	0.311	0.302	1.12E‐07	1.788	0.424	5.137
SST_min_	0.243	0.233	4.44E‐06	−1.869	7.738	12.452

We also used three multivariable methods to dissect the effects of IBD and IBE patterns on spatial genetic divergence. This analysis was greatly complicated by the multicollinearity among geographic factors and climatic factors. Using these multivariable methods, we found that *D*_csl_ and SST_max_ simultaneously explained most of the variation in genetic distance and that SST_max_ had a greater effect than did *D*_csl_ on genetic distance. In LASSO regression, only *D*_csl_ and SST_max_ had nonzero coefficients (0.679 and 0.283, respectively, Table 2). Similarly, *D*_*csl*_ and SST_max_ were the only two factors retained in the final GDM (for these two factors, the residual deviance of the response variable was 3.941 and 12.469, respectively) (Table S4). All indices produced by CCA indicated that *D*_csl_ and SST_max_ were the two most significant predictors (Figure 2c and Table S5). Although the Pratt measure and beta weight suggested that *D*_csl_ had higher importance than SST_max_, all other predictors revealed a more significant impact of SST_max_ (Figure 2d and Table S5).

**TABLE 2:** Regularized least squares (RLS) models indicating the relative importance of geographical and climatic factors.

Factors	Coeff.	SE	*t*	*p* value
LASSO				
Intercept	−0.06215	0.036556	−1.70020	0.093470
SSTmax	0	0.482180	0	1
SSTmed	0.679304	0.130626	5.200375	0.000002
SSTmin	0	0.354890	0	1
*D*_csl_	0.282812	0.317951	0.889483	0.376747
*D*_lat_	0	0.407032	0	1
*D*_gcc_	0	0.326984	0	1
Model	*R* ^2^	SSE	AIC	BIC
	0.766716	1.439215	−297.424	−280.927
Elastic net				
Intercept	−0.06007	0.036559	−1.64318	0.104768
SST_max_	0	0.482220	0	1
SST_med_	0.670278	0.130637	5.130855	0.000002
SST_min_	0	0.354919	0	1
*D*_csl_	0.286039	0.317978	0.899558	0.371397
*D*_lat_	0	0.407066	0	1
*D*_gcc_	0	0.327011	0	1
Model	*R* ^2^	SSE	AIC	BIC
	0.766678	1.439453	−297.411	−280.914

*Note*: RLS models were built using LASSO regression and elastic net regression. The columns in this table show the geographic/climatic factors used to build the models and the coefficient, standard error, *t* statistic, and *p* value of each factor. Each model is also summarized according to the coefficient of determination (*R* ^2^), SSE, AIC, and BIC.

Abbreviations: AIC, Akaike information criterion; BIC, ayesian information criterion; LASSO, least absolute shrinkage and selection operator; RLS, regularized least squares; SSE, sum of square errors.

We then divided the genome into PSRs and non‐PSRs (see Sections 2.6 and 3.5) and rebuilt a GDM for each division. The non‐PSR GDM was very similar to the whole‐genome GDM (Figure 2e,f). However, the magnitude of the total genetic distance change along *D*_csl_ and SST_max_ showed very different patterns in PSR‐specific GDM. Genetic differentiation within PSRs was largely affected by SST_max_, whereas *D*_csl_ had only a slight effect (Figure 2e,f).

### 3.4 Identification of temperature‐associated SNP loci and genes

**FIGURE 3:**
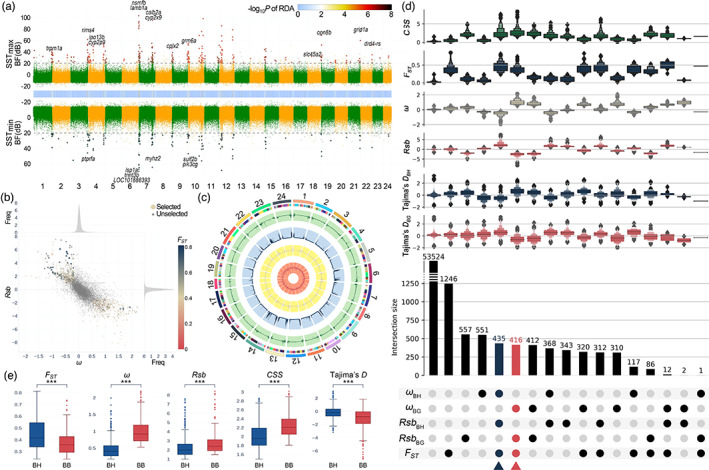
Genome‐wide selective signatures in the *L*. *maculatus* genome. (a) Manhattan plots showing the distribution of candidate SNPs associated with the SST_max_ (upper) and SST_min_ (lower) gradients. The intermediate track shows the ‐log_10_P‐value of all SNPs generated in the redundancy analysis (RDA). The larger red (SST_max_) and blue (SST_min_) dots in the Manhattan plots indicate the overlaps between RDA and the Bayesian approach. The positions of selected genes associated with candidate SNPs are indicated by their gene symbols. (b) Probability distributions of *F*_*ST*_, *ω*, and *Rsb* values over all sliding windows. Positively selected regions (PSRs) under natural selection are colored according to their *F*_*ST*_ values. (c) Circos plot showing the distributions of *CSS* (green), *F*_*ST*_ (blue), *ω* (khaki), and *Rsb* (red) values along all chromosomes. (d) UpSet plot showing intersections among the results of three selective signature detection algorithms. The columns of the matrix in the bottom panel correspond to five sets of outliers identified by different tests. The rows correspond to the intersections. For each row, the cells that form part of an intersection are filled in. Intersections identified as BH‐PSRs and BB‐PSRs are shown filled with blue and red, respectively. The bar plot in the middle panel indicates the number of outliers in each intersection. The strength of natural selection is shown in the boxplots in the top panel. (e) Statistically significant difference in the strength of natural selection between the BH‐ and BB‐PSRs. Five indicators, *CSS*, *F*_*ST*_, absolute *ω*, absolute *Rsb*, and *Tajima's D*, were used to measure the strength of natural selection. The symbol *** indicates that the observed *p* value is lower than 0.001.

We combined a Bayesian approach (BayPass) with RDA to detect SNPs associated with SST_max_ and SST_min_. BayPass revealed that 516 and 227 SNPs had strong associations solely with SST_max_ or SST_min_, respectively, and that 164 SNPs were associated with both SST_max_ and SST_min_ (Figure 3a and Table S6). The RDA detected 1927 outlier SNPs. A total of 334 SNPs were detected with both RDA and BayPass and identified as candidate temperature‐associated SNPs (178 SST_max_‐associated SNPs, 20 SST_min_‐associated SNPs, and 146 SNPs associated with both SST_max_ and SST_min_; Figure S5). We then searched genes located within 5 kbp of the candidate SNPs. In that search, we identified 186 SST_max_‐associated genes and 79 SST_min_‐associated genes (Figure 3a and Table S6). Functional enrichment revealed that the SST_max_‐associated genes were significantly enriched in 6 functional clusters, the top two of which were closely related to nervous system function (Figure S6 and Table S7). SST_min_‐associated genes were enriched in only three functional clusters (Figure S6 and Table S8). The enriched GO biological process, “muscle contraction” (GO:0006936), comprised four genes that encode components of muscle fibres and their binding proteins, proteins that have direct effects on locomotion. The observed enrichment of the “insulin signalling” pathway (WP1313) indicates that regulation of growth plays a role in the cold adaptation of BH populations

### 3.5 Candidate genomic regions under extreme positive selection

While Bayesian approaches are powerful for identifying SNP loci that harbour subtle shifts in allele frequency associated with environmental gradients, such approaches have difficulty detecting different haplotypes associated with individual genes (Salmón et al., 2021). Genome‐wide scanning for selective sweeps is a common and powerful way to identify candidate regions and genes that are subject to local adaptation and are involved in responses to climate change (Chen & Narum, 2021; Han et al., 2021; Li et al., 2021). Using this method, 436 and 416 PSRs were identified in the BH and BB populations, respectively (Figure 3, Figure S7, and Table S9). Approximately one‐third of the temperature‐associated SNPs were located on these PSRs. However, none of them were located within 1000 bp of the nearest PSR. The other two‐thirds of the temperature‐associated SNPs were distributed away from PSRs at distances ranging from 1000 bp to 10 Mbp (Figure S8). We noted that BB‐PSRs appeared to have experienced more intense natural selection than did BH‐PSRs. Stronger selective sweeps in BB‐PSRs were indicated by *ω*, *Rsb*, *Tajima's D*, and *CSS*, whereas higher *F*_ST_ suggested that the BH‐PSRs are more thoroughly differentiated (Figure 3e and Table S10). One reasonable explanation for this is that BB populations have a stronger but shorter selection history.

**FIGURE 4:**
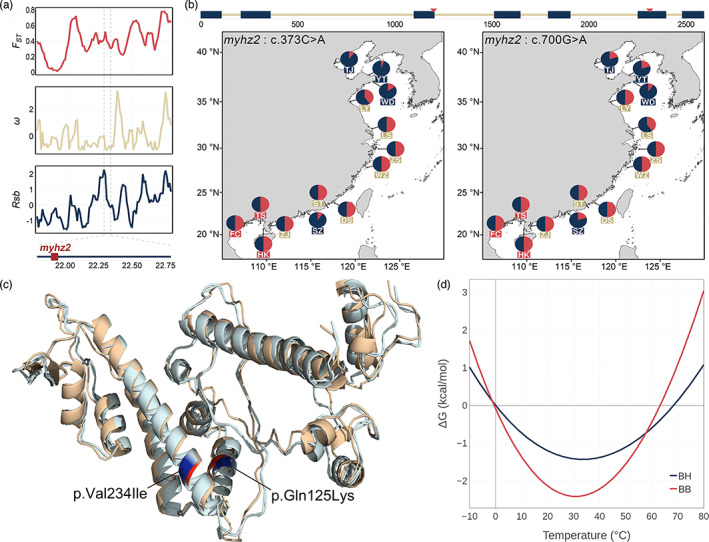
Selective signatures in fibre muscle components. (a) Three types of selective signatures around the myosin heavy chain (*myhz2*) gene. The red block in the bottom panel shows the genomic position of the *myhz2* gene. (b) Allele frequency of two nonsynonymous substitutions in the coding region of *myhz2* (c.373C > A, left; c700G > A, right) at each sampling site. The positions of the two substitutions in this gene are indicated by red arrows in the top panel. The blue blocks represent exons. (c) 3D protein structure of two types of myosin heavy chain proteins. The two substitutions are highlighted in blue (BH‐type) and red (BB‐type). (d) Gibbs free energy curve showing the thermal stability changes introduced by these two substitutions.

We identified 287 and 227 protein‐coding genes as BH‐ and BB‐PSGs, respectively (Tables S11 and S12). Within these genes, we identified 406 highly differentiated nonsynonymous SNPs (Table S13). Notably, many genes that encode essential structural components or regulatory proteins of striated muscle fibres were identified as PSGs in the BH population; these included *myhz2*, *titin*, and *cofilin‐2* (Figure 4a and Figure S9). We identified two nonsynonymous mutations (c.373C > A and c.700G > A) in *myhz2* that were highly differentiated between the BH and BB populations (Figure 4b). The former (c.373C > A) also has a significant association with SST_min_ (Bayes factor = 21.14; Figure 3a and Table S6). Although the tertiary structure of the myosin heavy chain did not change significantly in the presence of this mutation (Figure 4c), the thermal stability of this protein decreased in the BH population (Figure 4d and Table S14).

**FIGURE 5:**
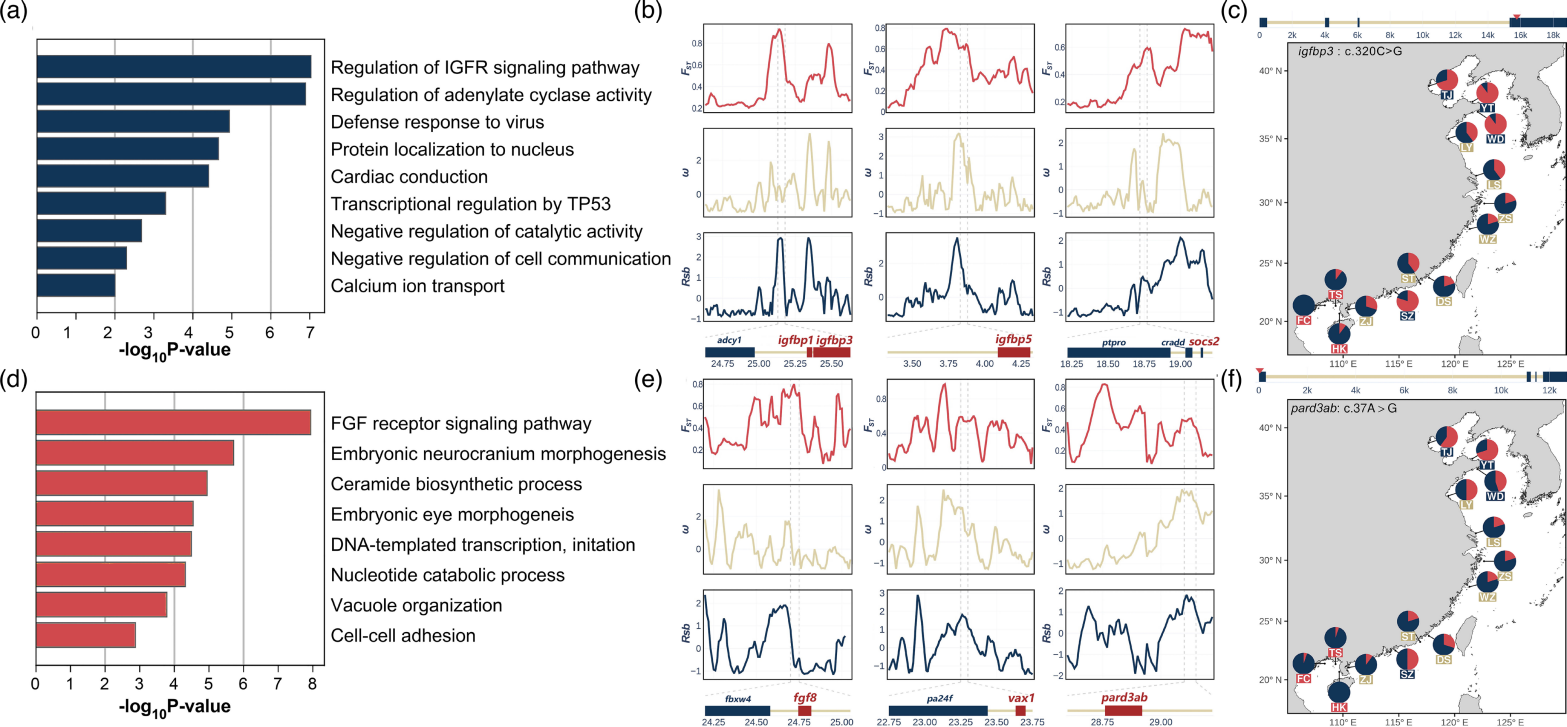
Functional category enrichment analysis and detection of selective signatures highlight different biological processes that may be responsible for cold and warm adaptation in the spotted sea bass. (a) Enriched functional categories identified using the BH‐PSGs. The categories are sorted according to the significance of their enrichment. (b) Selective signatures around some growth‐related genes. The bottom panel shows the genomic position of PSGs (the red blocks) and unselected nearby genes (the blue blocks). (c) Allele frequency of the nonsynonymous substitution in the coding region of *igfbp3* (c.320C > G) at each sampling site. The position of the substitution in this gene is indicated by a red arrow in the top panel. The blue blocks represent exons. (d) Enriched functional categories identified using the BB‐PSGs. The categories are sorted according to the significance of their enrichment. (e) Selective signatures around some visual system‐related genes. The bottom panel shows the genomic positions of PSGs (the red blocks) and unselected nearby genes (the blue blocks). (f) Allele frequency of the nonsynonymous substitution in the coding region of *pard3ab* (c.320C > G) at each sampling site. The position of the substitution in this gene is indicated by a red arrow in the top panel. The blue blocks represent exons.

We conducted overrepresentation analysis (ORA) and subsequent enrichment clustering to explore biological processes associated with climatic adaptation. As a result, we identified nine nonredundant enrichment clusters consisting of nine functional categories based on 34 BH‐PSGs (Figure 5a). The two most significant and largest clusters were represented by the GO terms “regulation of insulin‐like growth factor receptor signalling pathway” (GO:0043567) and “regulation of adenylate cyclase activity” (GO:0045761) and consisted of 10 nonredundant PSGs, the majority of which showed a clear association with growth hormone secretion and function (Figure 5b,c and Table S15). Thirty PSGs in the southernmost BB populations were enriched in eight functional categories, and these were further classified into eight enrichment clusters. Two relatively large clusters represented by the GO terms “ceramide biosynthetic process” (GO:0046513) and “embryonic eye morphogenesis” (GO:0048048) were directly related to the development and function of the visual system (Figure 5d); the genes in these clusters included *fgf8*, *vax1*, *pard3ab*, *foxg1*, and *ipo13*. The two most significant clusters, which were represented by the GO terms “fibroblast growth factor receptor signalling pathway” (GO:0008543) and “embryonic neurocranium morphogenesis” (GO: 0048702), contained a series of genes that are also probably instrumental in visual system development (Figure 5e,f, and Table S16).

## 4 DISCUSSION

### 4.1 Genetic structure and evolutionary history of spotted sea bass populations

The whole‐genome sequencing conducted in this study generated a vast number of SNPs, minimizing bias during the collection of genetic markers and making it possible to build an accurate genetic structure of the collected samples. Our results revealed three populations of sampled spotted sea bass (BH, IM, and BB) that differ greatly in their genetic compositions (Figure 1). Moreover, we observed that the habitats of these three populations are separated by the Qiongzhou Strait rather than by the brackish water present in large rivers. *L*. *maculatus* lives in nearshore waters and has limited migration ability (Sun et al., 2014) and wide salinity tolerance (Shao et al., 2018; Zhang et al., 2017); thus, it is less able to traverse geomorphological barriers such as peninsulas and shallow waterways than to spread across hydrochemical obstacles.

The Chinese sea bass is one of China's most important aquaculture species, ranking second in annual production among aquaculture fishes (China Fishery Statistical, 2021). Moreover, aquaculture enterprises continuously capture naturally spawned fry from the Bohai Gulf and the north Yellow Sea and transport them to farms in south China. The interbreeding and competition with wild populations caused by escape of fishes from aquaculture have long concerned researchers but have not yet been assessed. In this study, the five fish obtained from Shenzhen (SZ) near the main cultivation area might represent escaped individuals or their offspring; they share a genetic ancestry that is very similar to that of the BH individuals. If this is the case, it would indicate that aquaculture escapes have already severely impacted the population ecology of wild spotted sea bass (Figure 1c,d).

The evolutionary history of spotted sea bass populations has been illuminated by phylogenetic and demographic analyses. The spotted sea bass populations of Chinese offshore waters emerged in the northern area and then spread to the south until they reached the South China Sea. Approximately 50,000 years ago, BB populations uniquely suffered an intense population fluctuation during the Beiweitan II marine transgression and regression, and the last sizeable shoreline change events occurred in the South China Sea in the Middle Pleistocene (Zhaoshu Liu et al., 2002) (Figure 1e). During the marine transgression, the widening of the Qiongzhou Strait brought a greater chance of genetic communication between the IM and BB populations and thus dramatically inflated the BB populations. Subsequent marine regression severely evanished the entire Beibu Gulf and introduced a vigorous decrease in the effective size of the BB populations, which to date have still not recovered.

### 4.2 Geographic and climatic factors drive the genetic differentiation of spotted sea bass

IBD and IBE, which are two very common patterns of genetic differentiation, have not yet been examined in spotted sea bass. The results we obtained in this study revealed that both IBD and IBE patterns have significant effects on genetic differentiation in *L*. *maculatus*. It is worth noting that high collinearity was found between the most significant predictors in the IBD (*D*_csl_) and IBE (*D*_csl_) analyses (VIF = 9.767), and this brings a challenge to the dissection and comparison of the effectiveness of these two patterns. We employed three mutually independent methods, LASSO, GDM, and CCA, to address this issue. The results obtained using these three approaches agree that neither the effects of *D*_csl_ nor those of SST_max_ can be ignored when modelling the genetic differentiation of spotted sea bass (Figure 2 and Tables 1 and 2; Tables S4 and S5). The simultaneous and interwoven effects of geographic and environmental factors on the formation of genetic structure have been tested in various ectothermic animals. For example, Dionne et al. (2008) studied the genetic differentiation of Atlantic salmon and found that it is influenced by both coastal distance and temperature regime, whereas the influence of other factors such as latitude, river length, and altitude was not significant. A recent study revealed that geographic and climatic factors interact with each other and have complex effects on the population structure and speciation of Nearctic milksnakes (Burbrink et al., 2022). Furthermore, when building a GDM restricted to PSRs, we identified an expansion of the IBE effect as well as a contraction of the IBD effect (Figure 2e,f). Our results imply that the IBE effect was tightly linked to natural selection.

### 4.3 Selective signatures related to muscle fibre components

Bennett et al. conducted a series of comprehensive analyses based on which they proposed that extreme cold climates exert greater selection pressure than do hot climates (Bennett et al., 2021). In our results, all five selection signature statistics indicate that the BH populations of spotted sea bass have experienced more severe natural selection than the BB populations (Figure 3e). This implies that cold and warm adaptation of the spotted sea bass obeys Bennett's hypothesis.

Fish are less active in cold waters due to reduced muscle sensitivity caused by low temperatures (Riisgard & Larsen, 2009; Rome et al., 2000; Yan et al., 2012). In accordance with previous findings, many PSGs that encode essential structural components or regulatory proteins of striated muscle fibres were identified in the BH population (Figure 4a and Figure S9), indicating that changes in swimming ability may be a key behavioural adaptation process in the spotted sea bass. Among these genes, *myhz2* was repeatedly highlighted by selective signatures, association with SST_min_, and the presence of highly differentiated nonsynonymous mutations (Figures 3a and 4). Computational thermochemical analysis revealed that these mutations decrease the thermostability of the protein encoded by this gene. A previous study indicated that reduced thermostability of myosin is responsible for its more flexible structure in cold‐acclimated carp and that this reduced thermostability further results in a reduced activation enthalpy for contraction at low temperatures (Watabe, 2002). Such a reduction in the thermal stability of myofibrillar proteins has been reported as an adaptive mechanism that allows teleosts and other vertebrates to cope with environmental temperature changes (Howell et al., 1991; Rodgers et al., 1987; Takahashi et al., 2005). Overall, the evolutionary signatures of sarcomere‐related genes in BH populations of spotted sea bass revealed the presence of an adaptive mechanism for thermal acclimation that involves changes in intrinsic muscle contractile properties in teleosts.

### 4.4 Northern and southern populations may make different trade‐offs between growth and mortality

Our results show that the enrichment patterns of PSGs in the northern (BH) and southern (BB) populations differ greatly. The most significantly enriched functional category of BH‐PSGs, “regulation of insulin‐like growth factor (IGF) receptor signalling pathway”, included four (*igfbp1*, *igfbp2b*, *igfbp3*, and *igfbp5*) of the five Igfbp genes (Figure 5a–c and Figure S10). IGFBPs are a series of important regulators of a powerful and complex system that regulates the growth of various organisms and tissues—the growth hormone (GH)/IGF‐1 axis (Pérez‐Sánchez et al., 2018). Various studies have indicated that IGFBPs play a crucial role in the growth of the skeletal, muscular, and nervous systems in teleosts (Shimizu & Dickhoff, 2017). In addition, we identified selective signatures of the suppressor of the cytokine signalling‐2 (*socs‐2*) gene, an important negative regulator of the GH/IGF‐1 axis. The product of this gene can competitively bind to Igf‐1r and thereby impede the function of IGF‐1 in various species (Greenhalgh et al., 2002; Horvat & Medrano, 2001; Liu et al., 2006; Philip & Vijayan, 2015).

In the BB population, both the Bayesian and selective signature scanning approaches identified PSGs that are closely related to the development and function of the nervous system (Figures 3a and 5d–f; Tables S7 and S16). Notable among them were genes related to visual sensing and functional categories (Figures 5d–f and S11; Table S16). Although other types of sensation are involved, visual perception plays a crucial role by allowing organisms to detect predators and prey (McCormick & Manassa, 2008; Oliveira et al., 2017; Puvanendran et al., 2002). In tropical zones, predator–prey interactions are stronger and more complex than those in colder areas (Schemske et al., 2009). In response to this, fishes generate a variety of alterations in their behaviour, physiology, and demography (Culumber, 2020; Mennen & Laskowski, 2018; Mitchell & Harborne, 2020; Xu et al., 2019).

The divergent patterns of PSG enrichment found in the two populations suggest that the populations may adopt different strategies that involve different trade‐offs between growth and mortality. It has been demonstrated that a higher capacity for growth is a common adaptation to shorter growing seasons in high‐latitude environments (Angilletta et al., 2002; Conover & Schultz, 1995; Suzuki et al., 2010). However, rapid growth is realized through frequent feeding and is usually accompanied by a high risk of predation, a situation that has been termed the “growth‐mortality trade‐off” (Lankford et al., 2001; Stocks et al., 2005; Suzuki et al., 2010). A common garden experiment demonstrated that northern populations of *L*. *maculatus* had a significantly higher growth rate (2.4 g/d) than their southern conspecifics (1.7 g/d) (Chen et al., 2001). However, the presence of a latitudinal difference in predator avoidance remains to be validated in this species.

It is worth noting that dissection of the interactions of the genome of an organism with the environment is very complicated. The presence of relatively sparse sampling sites and multicollinearity among geographic and environmental factors undoubtedly increase the challenge. Therefore, the findings of this study should be validated by quantitative studies of adaptive traits followed by common garden‐variety experiments in the future.

## CONFLICT OF INTEREST STATEMENT

The authors declare that they have no conflict of interest. The authors alone are responsible for the content and writing of the article.

## Supporting information


Appendix S1

Table S1

Figure S1

## Data Availability

All genomic resequencing data of *L*. *maculatus* are openly available in the Sequence Read Archive database of the National Center for Biotechnology Information at https://www.ncbi.nlm.nih.gov/bioproject/PRJNA701455, NCBI BioProject Accession: SRP305917. The improved reference genome assembly and annotation are openly available in figshare at https://doi.org/10.6084/m9.figshare.7405694.v2, and in the Genome Warehouse in National Genomics Data Center, under accession number GWHCAYQ00000000 that is publicly accessible at https://ngdc.cncb.ac.cn/gwh/Assembly/GWHCAYQ00000000.
