# Supplementary material for: Genome‐wide evolutionary signatures of climate adaptation in spotted sea bass inhabiting different latitudinal regions
Source: Evol Appl. 2023 Apr 27;16(5):1029–43. doi: 10.1111/eva.13551 (PMC10197228; doi:10.1111/eva.13551)
Supplement: Supplementary file 1 — Appendix S1 [file EVA-16-1029-s002.docx]

**Supplemental Information for:**

**Genome-wide evolutionary signatures of climate adaptation in spotted sea bass inhabiting different latitudinal regions**

**Table of Contents:**

| Improvement of chromosome-level scaffolding of spotted sea bass reference genome | Page 2 |
| --- | --- |
| Gene prediction and annotation of the upgraded genome | Page 3 |
| Comparative genomics analysis between the two versions of genome | Page 4 |
| Data pre-processing, variant calling and filtering | Page 5 |
| References | Page 6 |
| Supplemental Information Figures | Page 8 |
| Supplemental Information Tables | Page 11 |

**Improvement of chromosome-level scaffolding of spotted sea bass (*Lateolabrax maculatus*) reference genome**

Our previous reference genome assembly was completed with high accuracy and contiguity at the scaffold level (GenBank accession: GCA_004028665.1) (B. H. Chen et al., 2019). However, an obsoleted software, LACHESIS, was used in processing the raw Hi-C data and chromosome-level scaffolding, which may have introduced sub-optimal orderings and orientations of scaffolds (Zhang, Zhang, Zhao, Ming, & Tang, 2019). Hence, we refined our previous reference genome at the chromosome level using a critically acclaimed software, 3D-DNA (Dudchenko et al., 2017).

We used our previously obtained scaffold-level genome assembly, 163.3 Gb raw high-throughput chromosome conformation capture (Hi-C) reads, which had been uploaded into the NCBI Genome Assembly database (Accession ID: ASM402866v1), and the NCBI Short Reads Archive database (Accession ID: SRR8240566), respectively. Raw reads were filtered out by SolexaQA++ (version v.3.1.7.1(Dionne, Caron, Dodson, & Bernatchez, 2008)) if the proportion of uncertain bases exceeded 10% of the total length, the proportion of low-quality bases exceeded 50% of the total length, or the reads contained sequencing adaptors. First, we used Juicer (version 1.5.5) (Durand, Shamim, et al., 2016) to transform raw sequencing data into a list of Hi-C contacts (pairs of genomic positions that were adjacent to each other in 3D space during the experiment). Then, we employed the haploid mode of 3D-DNA pipeline (version 201008) (Dudchenko et al., 2017) to generate a candidate chromosome-level assembly. The threshold of scaffold size was set to 15000. The number of rounds for misjoin correction were set to 2. Five steps, polish, split, seal, merge, and finalize, were performed automatically with default parameters using the “run-asm-pipeline” script provided by 3D-DNA. Finally, the candidate assembly was reviewed and finalized manually based on the associated contact map using Juicebox (version 1.5.3) (Durand, Robinson, et al., 2016). The improved genome assembly contained 480 scaffolds, a total length of 589.99 Mbp, showing higher integrity than the previous assembly. The integration level was also slightly improved to 98.76% (**SI Figure 1 and SI Table 1**).

**Gene prediction and annotation of the upgraded genome**

The repeat sequence library detected from the original genome sequences (B. Chen et al., 2019) were used to annotated and mask repetitive sequences in the ungraded genome. Then, Braker2 (version 2.1.5) (Bruna, Hoff, Lomsadze, Stanke, & Borodovsky, 2021), a fully automated gene prediction pipeline was employed to produce evidence to gene structures based on protein sequences of closely related species, which were downloaded from the Ensembl database (release 104), including *Oreochromis niloticus* (O_niloticus_UMD_NMBU), *Takifugu rubripes* (FUGU5),  *Danio rerio* (GRCz11), *Larimichthys crocea* (L_crocea_2.0), *Cynoglossus semilaevis* (Cse_v1.0), *Oryzias latipes* (GCA_002234675.1), *Gasterosteus aculeatus* (BROAD S1), and  *Lates calcarifer* (ASB_HGAPassembly_v1). In addition, RNA-seq reads generated from a pooled cDNA library of 12 tissues (NCBI SRA accession IDs: SRR6041051 and SRR6041052) were mapped to the updated genome using Blat (version 420) (Kent, 2002), GMAP (version 2021-08-25) (Wu & Watanabe, 2005) and HISAT2 (version 2.2.1) (Kim, Paggi, Park, Bennett, & Salzberg, 2019). The HISAT2 alignments were used to generate a *de novo* transcript assembly by employing Trinity (version 2.13.0) (Grabherr et al., 2011). The software PASA (version v2.5.0) (Haas et al., 2003) were used to a comprehensive transcript assembly. Then, Augustus and GeneMark were employed for *de novo* gene prediction based on a training set generated by transdecoder (version 5.5.0) with the PASA transcripts. Finally, the gene models generated by Augustus and GeneMark, the PASA transcripts, the Transdecoder training set, and the spliced transcript alignment generated by BLAT and GMAP were integrated into a consensus gene set using the EvidenceModeler software (version 1.1.1) (Haas et al., 2008). The resulting protein sequences were subsequently annotated based on the NCBI nr and SwissProt protein databases. Gene ontology (GO) and Kyoto Encyclopedia of Genes and Genomes (KEGG) annotations of genes were assigned using the InterProScan program (version 5.26) (Jones et al., 2014) and the KEGG Automatic Annotation Server (KAAS, version sa2_140224) (Moriya, Itoh, Okuda, Yoshizawa, & Kanehisa, 2007), respecitvely.

**Comparative genomics analysis between the two versions of genome**

To compare the correctness of the refined (denoted as 3D) and original (denoted as LAC) chromosome-level scaffolding, we conducted a comparative genomic analysis among the new and old assembly using three different references: a high-density linkage map of this species (Liu et al., 2020) and chromosome assemblies of two related species, the Asian seabass (*Lates calcarifer*) (Vij et al., 2016) and the mandarin fish (*Siniperca chuatsi*). The three references were denoted as LM (the linkage map), AS (the reference genome of Asian sea bass) and MF (the reference genome of mandarin fish) for concision hereafter. Firstly, we blat the flanking sequences of SNPs in the linkage map against our two versions of genome assemblies. Our two versions of genomes were aligned with the two related species’ genomes using Mummer4 (version 4.0.0beta2) (Marcais et al., 2018) with default parameters. All alignments with identities lower than 90% and lengths shorter than 1000 bp we filtered out. Then, Spearman’s rank correlation coefficients (ρ_S_) were employed to indicate the order similarity of genetic markers and alignments between each query-reference pair. Then we conducted a one-way analysis of variance (ANOVA) to assess the significance of ρ_S_ in each comparison. Spearman’s rank correlation coefficients and one-way ANOVA were calculated using an in-house Python script with some basic statistic functions imported from the “SciPy” library.

The mean ρ_S_ values between 3D and three references (LM, MF and AS) were 0.882, 0.827 and 0.694, respectively. The three values were much lower (0.639, 0.621 and 0.528) between LAC and the references (**SI Figure 2 and SI Table 2**). The differences in ρ_S_ values between 3D and LAC were significant when taking LM and MF as references. Moreover, an Oxford plot shows that 3D has much fewer truncations and inversions than LAC compared to the linkage map (**SI Figure 3**). These results show that the new assembly showed increased collinearities with all references, providing an accurate reference genome for the downstream population genomics and genome evolution studies. The improved reference genome assembly has been updated in GenBank (accession ID: GCA_004028665.2).

**Read pre-processing, variant calling and filtering**

The quality of raw sequencing data was firstly assessed and controlled using SolexaQA++ (version 3.1.1) (Cox, Peterson, & Biggs, 2010). During this step, reads containing adaptor sequences, with a proportion of uncertain or low-quality bases exceeding 10%, and with lengths shorter than 50 bp were removed. Then, the retained reads was aligned to the refined genome using the BWA-MEM algorithm (version 0.7.17-r1188) (Li & Durbin, 2009) with default parameters. Subsequently, variant calling was performed using GATK (version 4.0.2.1) (McKenna et al., 2010) under the guidance of GATK Best Practices (Van der Auwera et al., 2013) were applied for variant detection. The "SortSam" and "MarkDuplicates" implements in Plink were used to sort the alignments and mark the PCR duplicates. Detection of single-sample variants was performed using "HaplotypeCaller". The "GenomicsDBImport" and "GenotypeGVCFs" were used for joint genotyping. Since there were no genome-wide known sites or genotyped data in the *L. maculatus* genome, hard filtering was applied on this callset. SNPs with less ideal quality were marked using "VariantFiltration" in GATK according to the following threshold: "QD < 2.0 || QUAL < 30.0 || SOR > 3.0 || FS > 60.0 || MQ < 40.0 || MQRankSum < -12.5 || ReadPosRankSum < -8.0". Then, the "SelectVariant" program in the same software was used to exclude all InDels and SNPs marked in the previous step. An extra step of filtering was applied using VCFtools (version 0.1.15) (Danecek et al., 2011) to remove SNPs with multiple alleles, a minor allele count less than 2, a genotype missing counts greater than 2 or a minor allele frequency (MAF) less than 0.05. At last, the final SNP set was annotated with SnpEff (version 4.3t) (Cingolani et al., 2012).

**Data Visualization**

Most subfigures were made using the powerful Python graphing library “Plotly” (version 5.3.0) (https://plotly.com/python/). However, there were some exceptions. **Fig. 1a** was plotted with two R libraries, “ggmap” (version 3.0.0) for plotting the background map and “ggplot2” (version 3.3.5) for coloring sea area according to the temperatures and for adding labels. **Fig. 1b** was plottted using “ggplot2”. FigTree (version 1.4.4) was used to visualize and beautify the phylogenetic tree in **Fig. 1c**. **Fig. 3c** was created with Circos (version 0.69-8), a Perl software package. We employed two Python libraries, “upsetplot” (version 0.4.1) and “matplotlib” (version 3.3.3) to generate **Fig. 3d**. When making **Fig. 4b**, **Fig. 5c**, and **Fig. 5f**, we used “ggmap” (version 3.0.0) to plot the background map and used “scatterpie” (version 0.1.7) to add the pie plots onto the maps. **Fig. 5a** and **Fig. 5d** were output files of the webtool “Metascape”. All Python, R, and Bash scripts used to connect the above libraries and tools were written by ourselves.

**References**

Bruna, T., Hoff, K. J., Lomsadze, A., Stanke, M., & Borodovsky, M. (2021). BRAKER2: automatic eukaryotic genome annotation with GeneMark-EP plus and AUGUSTUS supported by a protein database. *Nar Genomics and Bioinformatics, 3*(1). doi:ARTN lqaa108

10.1093/nargab/lqaa108

Chen, B., Li, Y., Peng, W., Zhou, Z., Shi, Y., Pu, F., . . . Xu, P. (2019). Chromosome-Level Assembly of the Chinese Seabass (Lateolabrax maculatus) Genome. *Front Genet, 10*, 275. doi:10.3389/fgene.2019.00275

Chen, B. H., Li, Y., Peng, W. Z., Zhou, Z. X., Shi, Y., Pu, F., . . . Xu, P. (2019). Chromosome-Level Assembly of the Chinese Seabass (Lateolabrax maculatus) Genome. *Frontiers in Genetics, 10*. doi:ARTN 275

10.3389/fgene.2019.00275

Cingolani, P., Platts, A., Wang, L. L., Coon, M., Nguyen, T., Wang, L., . . . Ruden, D. M. (2012). A program for annotating and predicting the effects of single nucleotide polymorphisms, SnpEff: SNPs in the genome of Drosophila melanogaster strain w(1118); iso-2; iso-3. *Fly, 6*(2), 80-92.

Cox, M. P., Peterson, D. A., & Biggs, P. J. (2010). SolexaQA: At-a-glance quality assessment of Illumina second-generation sequencing data. *BMC Bioinformatics, 11*.

Danecek, P., Auton, A., Abecasis, G., Albers, C. A., Banks, E., DePristo, M. A., . . . Genomes Project Analysis, G. (2011). The variant call format and VCFtools. *Bioinformatics, 27*(15), 2156-2158. doi:10.1093/bioinformatics/btr330

Dionne, M., Caron, F., Dodson, J. J., & Bernatchez, L. (2008). Landscape genetics and hierarchical genetic structure in Atlantic salmon: the interaction of gene flow and local adaptation. *Molecular Ecology, 17*(10), 2382-2396.

Dudchenko, O., Batra, S. S., Omer, A. D., Nyquist, S. K., Hoeger, M., Durand, N. C., . . . Aiden, E. L. (2017). De novo assembly of the Aedes aegypti genome using Hi-C yields chromosome-length scaffolds. *Science, 356*(6333), 92-95. doi:10.1126/science.aal3327

Durand, N. C., Robinson, J. T., Shamim, M. S., Machol, I., Mesirov, J. P., Lander, E. S., & Aiden, E. L. (2016). Juicebox Provides a Visualization System for Hi-C Contact Maps with Unlimited Zoom. *Cell Systems, 3*(1), 99-101. doi:10.1016/j.cels.2015.07.012

Durand, N. C., Shamim, M. S., Machol, I., Rao, S. S. P., Huntley, M. H., Lander, E. S., & Aiden, E. L. (2016). Juicer Provides a One-Click System for Analyzing Loop-Resolution Hi-C Experiments. *Cell Systems, 3*(1), 95-98. doi:10.1016/j.cels.2016.07.002

Grabherr, M. G., Haas, B. J., Yassour, M., Levin, J. Z., Thompson, D. A., Amit, I., . . . Regev, A. (2011). Full-length transcriptome assembly from RNA-Seq data without a reference genome. *Nat Biotechnol, 29*(7), 644-652. doi:10.1038/nbt.1883

Haas, B. J., Delcher, A. L., Mount, S. M., Wortman, J. R., Smith, R. K., Hannick, L. I., . . . White, O. (2003). Improving the Arabidopsis genome annotation using maximal transcript alignment assemblies. *Nucleic acids research, 31*(19), 5654-5666.

Haas, B. J., Salzberg, S. L., Zhu, W., Pertea, M., Allen, J. E., Orvis, J., . . . Wortman, J. R. (2008). Automated eukaryotic gene structure annotation using EVidenceModeler and the program to assemble spliced alignments. *Genome Biology, 9*(1).

Jones, P., Binns, D., Chang, H. Y., Fraser, M., Li, W. Z., McAnulla, C., . . . Hunter, S. (2014). InterProScan 5: genome-scale protein function classification. *Bioinformatics, 30*(9), 1236-1240.

Kent, W. J. (2002). BLAT - The BLAST-like alignment tool. *Genome Research, 12*(4), 656-664.

Kim, D., Paggi, J. M., Park, C., Bennett, C., & Salzberg, S. L. (2019). Graph-based genome alignment and genotyping with HISAT2 and HISAT-genotype. *Nature Biotechnology, 37*(8), 907-+.

Li, H., & Durbin, R. (2009). Fast and accurate short read alignment with Burrows-Wheeler transform. *Bioinformatics, 25*(14), 1754-1760. doi:10.1093/bioinformatics/btp324

Liu, Y., Wang, H. L., Wen, H. S., Shi, Y., Zhang, M. Z., Qi, X., . . . Li, Y. (2020). First High-Density Linkage Map and QTL Fine Mapping for Growth-Related Traits of Spotted Sea bass (Lateolabrax maculatus). *Marine Biotechnology, 22*(4), 526-538.

Marcais, G., Delcher, A. L., Phillippy, A. M., Coston, R., Salzberg, S. L., & Zimin, A. (2018). MUMmer4: A fast and versatile genome alignment system. *Plos Computational Biology, 14*(1). doi:ARTN e1005944

10.1371/journal.pcbi.1005944

McKenna, A., Hanna, M., Banks, E., Sivachenko, A., Cibulskis, K., Kernytsky, A., . . . Daly, M. (2010). The Genome Analysis Toolkit: a MapReduce framework for analyzing next-generation DNA sequencing data. *Genome Research, 20*(9), 1297-1303.

Moriya, Y., Itoh, M., Okuda, S., Yoshizawa, A. C., & Kanehisa, M. (2007). KAAS: an automatic genome annotation and pathway reconstruction server. *Nucleic acids research, 35*, W182-W185.

Van der Auwera, G. A., Carneiro, M. O., Hartl, C., Poplin, R., Del Angel, G., Levy-Moonshine, A., . . . DePristo, M. A. (2013). From FastQ data to high confidence variant calls: the Genome Analysis Toolkit best practices pipeline. *Curr Protoc Bioinformatics, 43*, 11 10 11-33. doi:10.1002/0471250953.bi1110s43

Vij, S., Kuhl, H., Kuznetsova, I. S., Komissarov, A., Yurchenko, A. A., Van Heusden, P., . . . Orban, L. (2016). Chromosomal-Level Assembly of the Asian Seabass Genome Using Long Sequence Reads and Multi-layered Scaffolding. *PLoS Genet, 12*(4), e1005954. doi:10.1371/journal.pgen.1005954

Wu, T. D., & Watanabe, C. K. (2005). GMAP: a genomic mapping and alignment program for mRNA and EST sequences. *Bioinformatics, 21*(9), 1859-1875.

Zhang, X., Zhang, S., Zhao, Q., Ming, R., & Tang, H. (2019). Assembly of allele-aware, chromosomal-scale autopolyploid genomes based on Hi-C data. *Nat Plants, 5*(8), 833-845. doi:10.1038/s41477-019-0487-8

**Supplemental Information Figures**


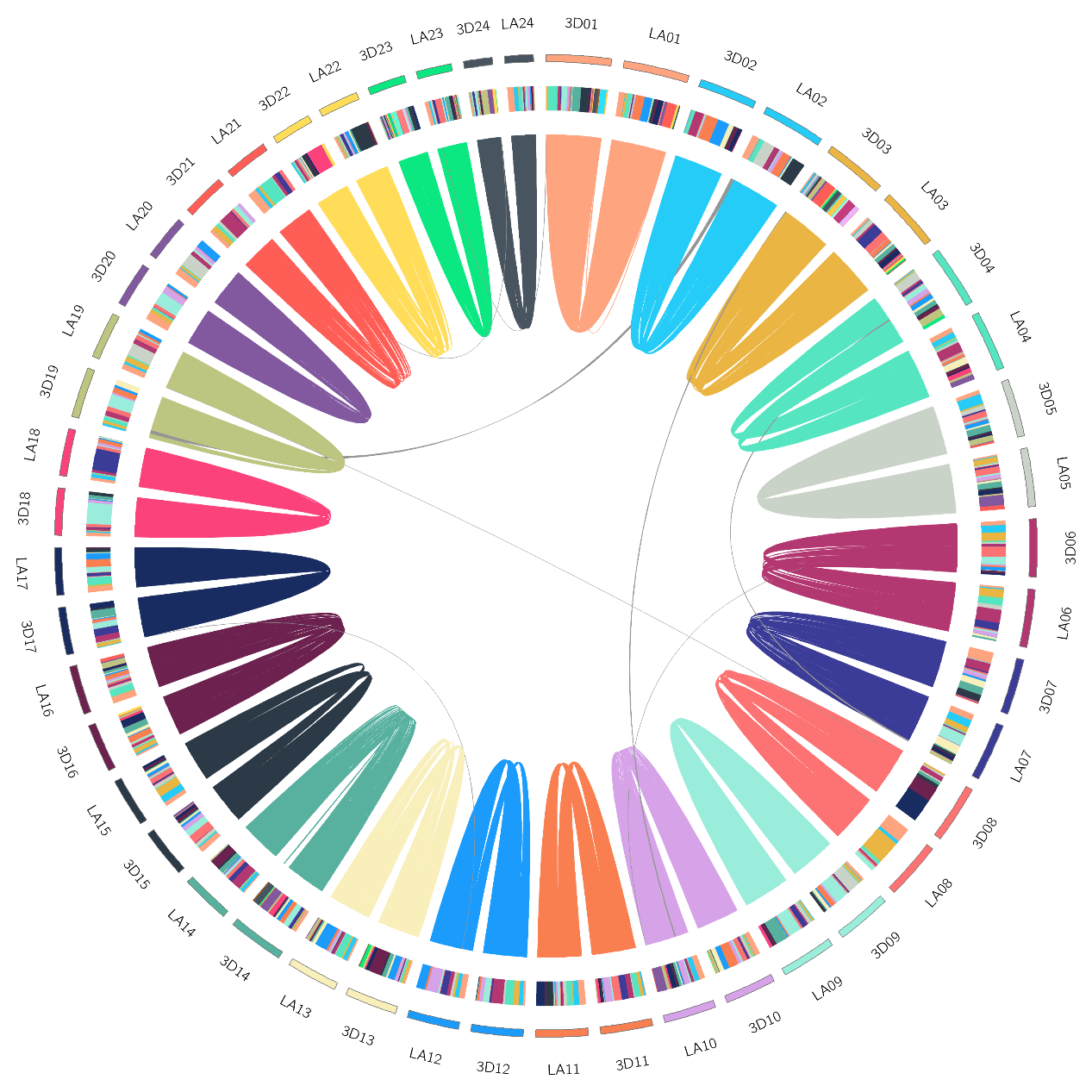


**SI Figure 1. Refined Chromosome assembly of spotted sea bass and genomic collinearity between the new (right) and old (left) assemblies.** The outer track visualizes the individual chromosomes. The intermediate indicates how scaffolds were assembled into chromosomes. The inner track


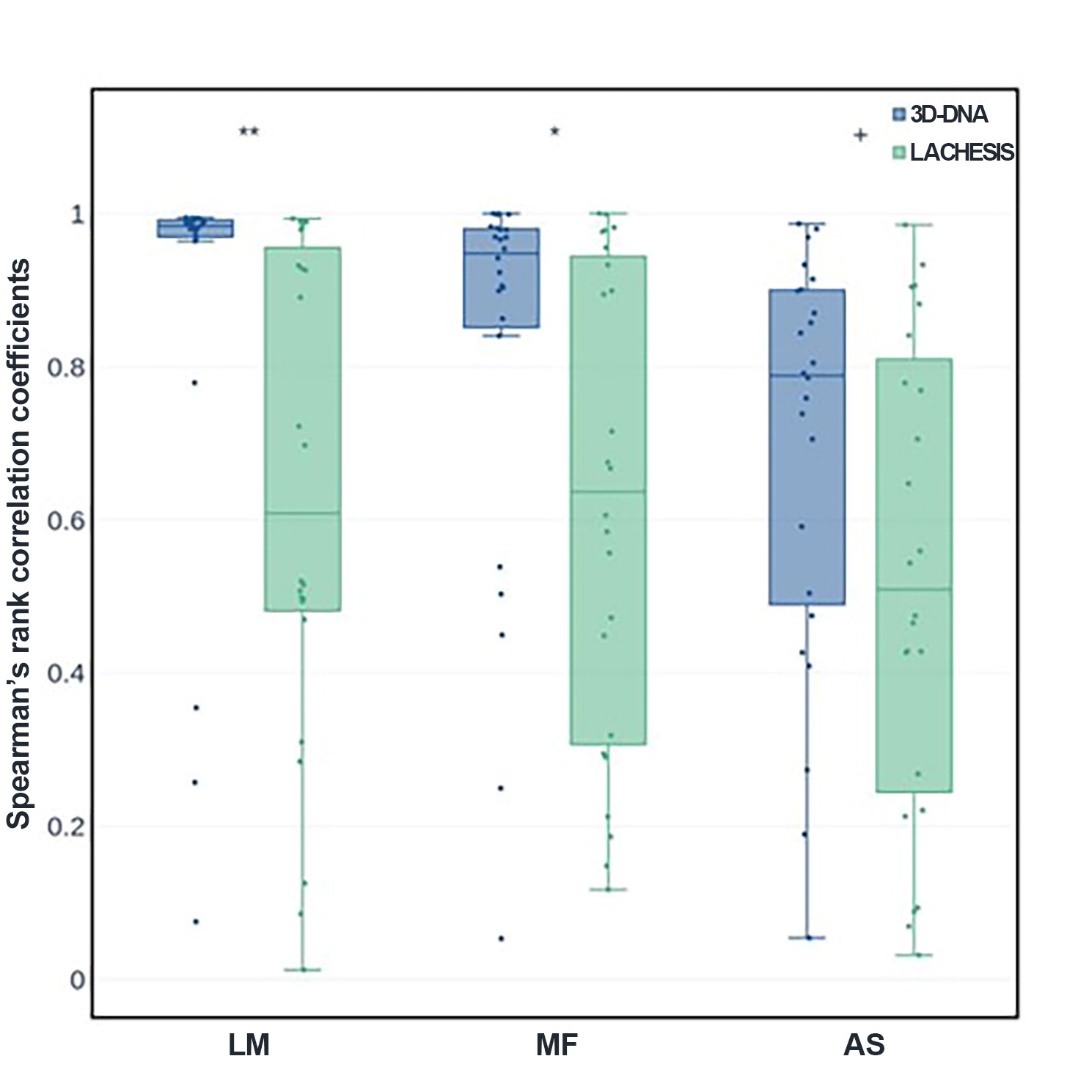


**SI Figure 2. New chromosome-level assembly employing 3D-DNA shows higher colinearity to references**

The Spearman’s rank correlation coefficient was employed to indicate the colinearity between genome assembly of the spotted sea bass and the linkage map of this species (LM)/the genome of the mandarin fish (MF)/ the genome of the Asian sea bass (AS)


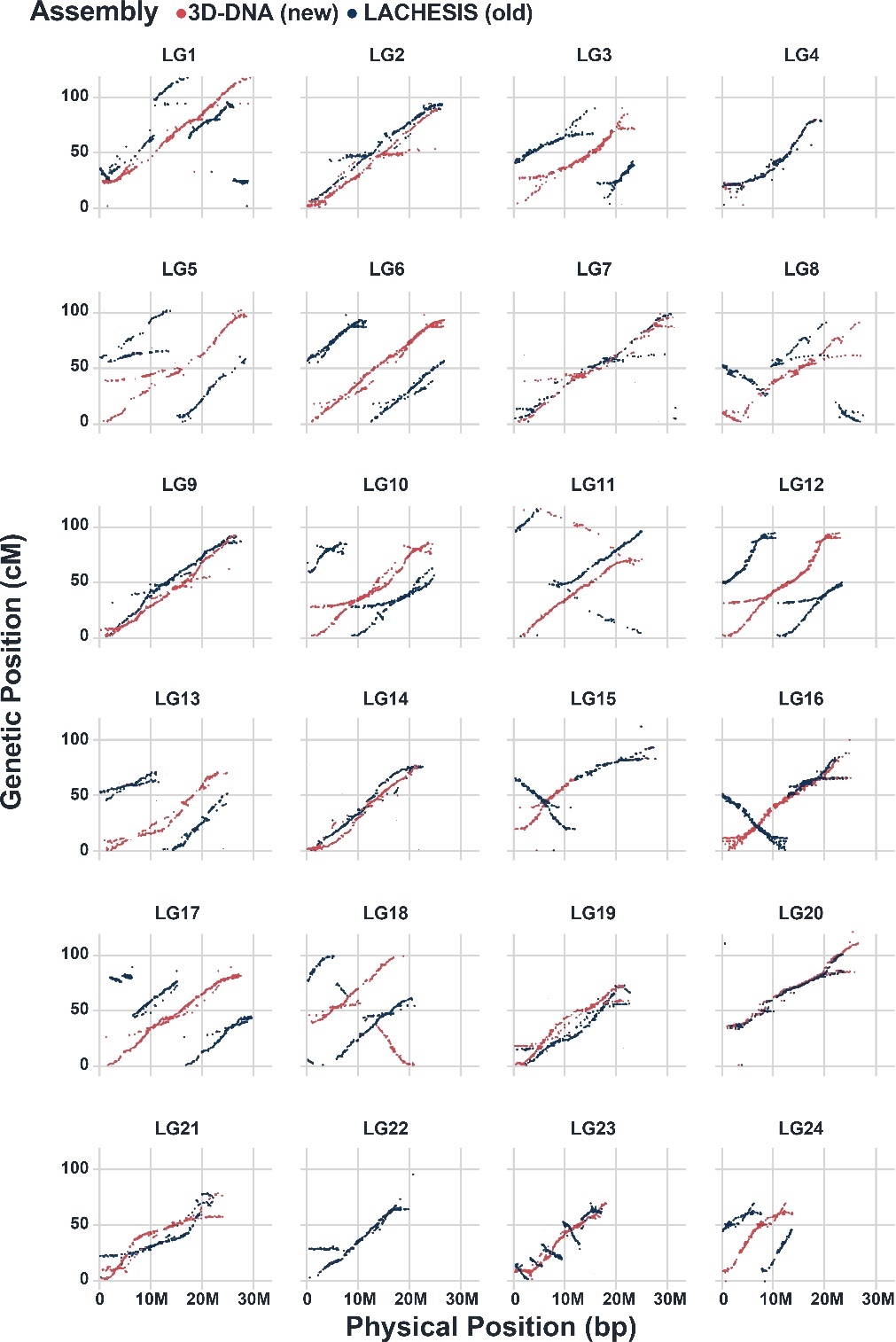


**SI Figure 3. A scatter plot shows the improvement in the collinearity with the linkage map of the new assembly from the old one**

Each SNP on the linkage map was plotted as a pair of dots. The red dot indicated its position in the new version genome (assembled using 3D-DNA), while the blue dot indicated its position in the old genome assembled using LACHESIS

**upplemental Information Tables**

**SI Table 1. Basic information of two versions of chromosome assemblies**

|  | **3D-DNA** | **LACHESIS** |
| --- | --- | --- |
| Total Length (TL) | 589.99 | 586.07 |
| TL_chrs_/TL_scaffolds_^*^ | 0.9876 | 0.9811 |
| Number of Chromosomes | 24 | 24 (manually set) |

^*^: Ratio between the total length of all chromosomes and that of all scaffolds.

**SI Table 2. Spearman’s rank correlation coefficients (ρ_S_) indicate the improvement of chromosome-level scaffolding**

|  | **3D** | | **LACHESIS** | |
| --- | --- | --- | --- | --- |
|  | **Mean ρ^a^** | **Mean P-value (-log10 transfromed)** | **Mean ρ^a^** | **Mean P-value (-log10 transfromed)** |
| LM^a^ | 0.882^**^ | 190.847^***^ | 0.639^**^ | 78.531^***^ |
| MF^b^ | 0.827^*^ | 109.26^*^ | 0.621^*^ | 51.605^*^ |
| AS^c^ | 0.694^+^ | 39.300^+^ | 0.528+ | 25.885^+^ |

^a^: The linkage map of the spotted sea bass was taken as the reference.

b: The genome of the mandarin fish was taken as the reference.

^c^: The genome of the Asian sea bass was taken as the reference.
