# Supplementary material for: Genome‐wide evolutionary signatures of climate adaptation in spotted sea bass inhabiting different latitudinal regions
Source: Evol Appl. 2023 Apr 27;16(5):1029–43. doi: 10.1111/eva.13551 (PMC10197228; doi:10.1111/eva.13551)
Supplement: Supplementary file 3 — Figure S1 [file EVA-16-1029-s003.docx]

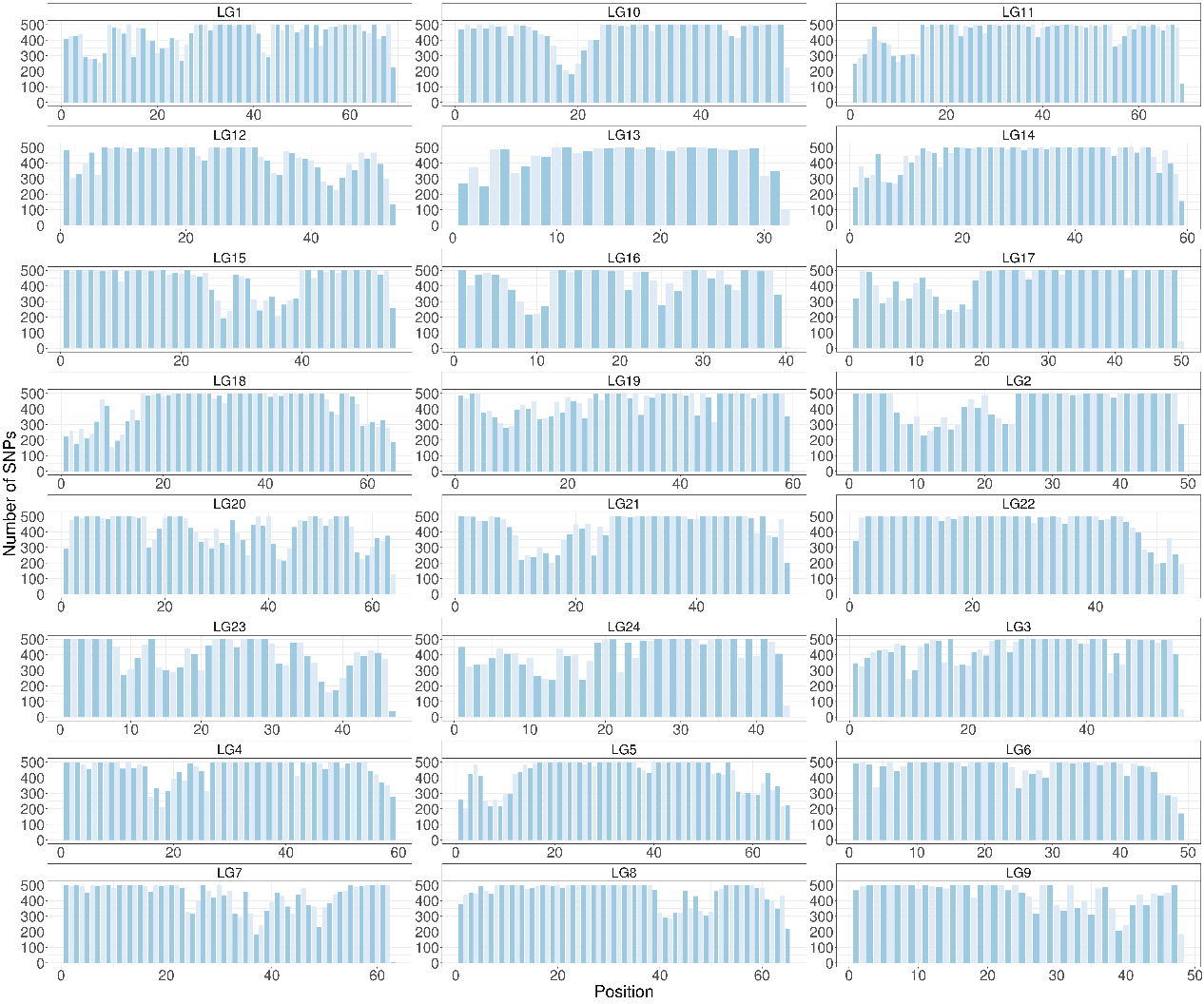


**Figure S1. SNP distribution along chromosomes of *L. maculatus* genome.**

The number of SNPs was calculated within each 1-Mb bin.


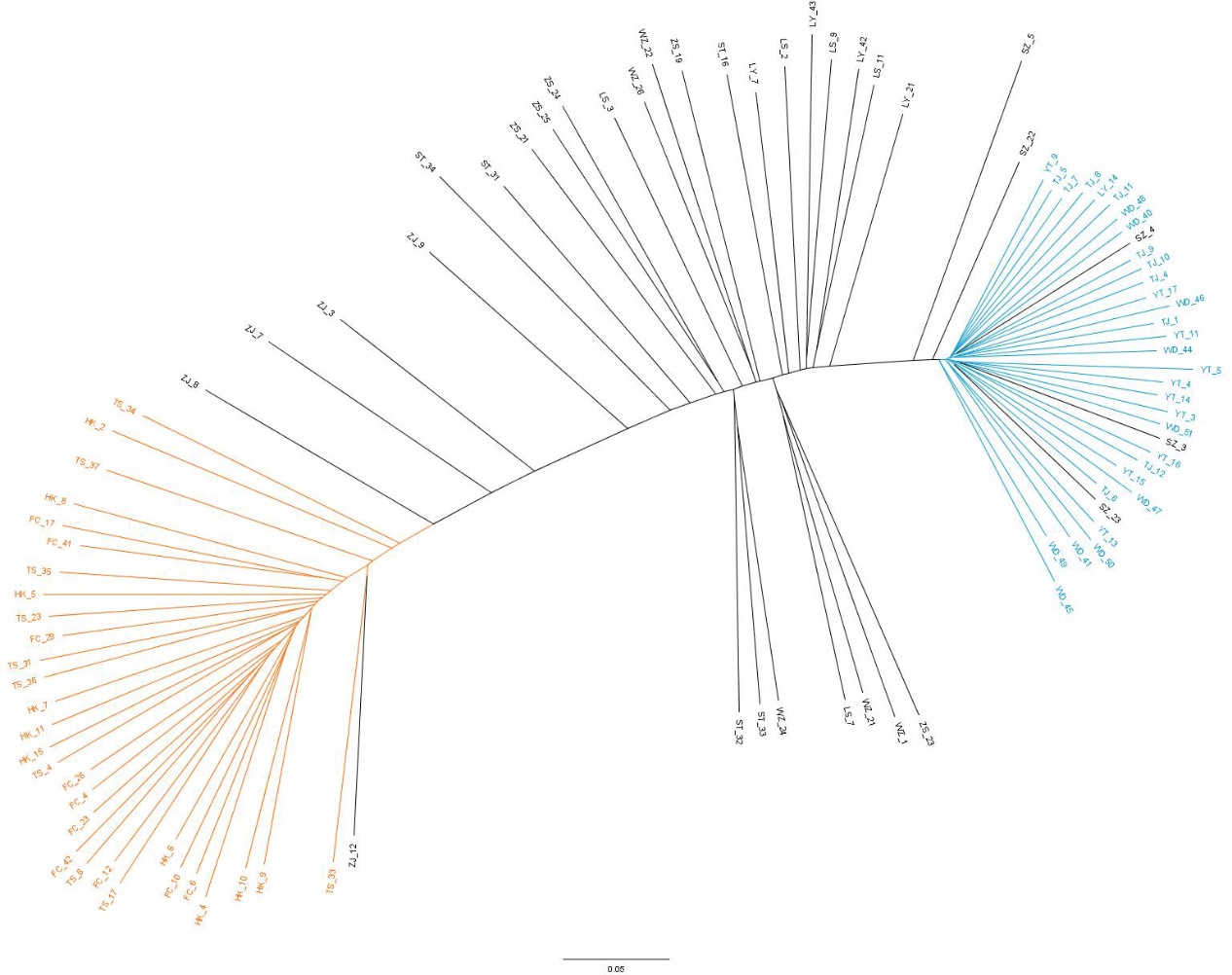


**Figure S2. An unrooted neighbor-joining tree of all sequenced fish.**

The Beibu Gulf population (BB), Bohai Gulf population (BH), and intermediate population (IM) are colored orange, blue and black.


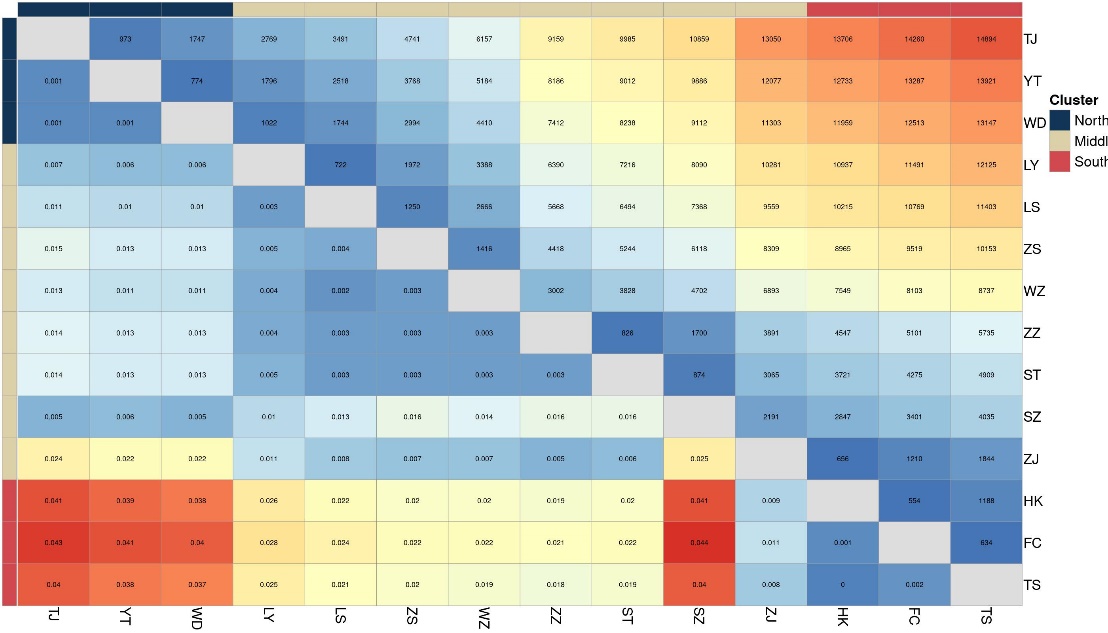


**Figure S3. A heatmap shows pairwise genetic distances and distances along coastlines (D_csl_) among populations.**

The pairwise genetic distances were placed in the bottom-left triangular matrix, and the pairwise D_csl_ were plotted in the top-right triangular matrix.


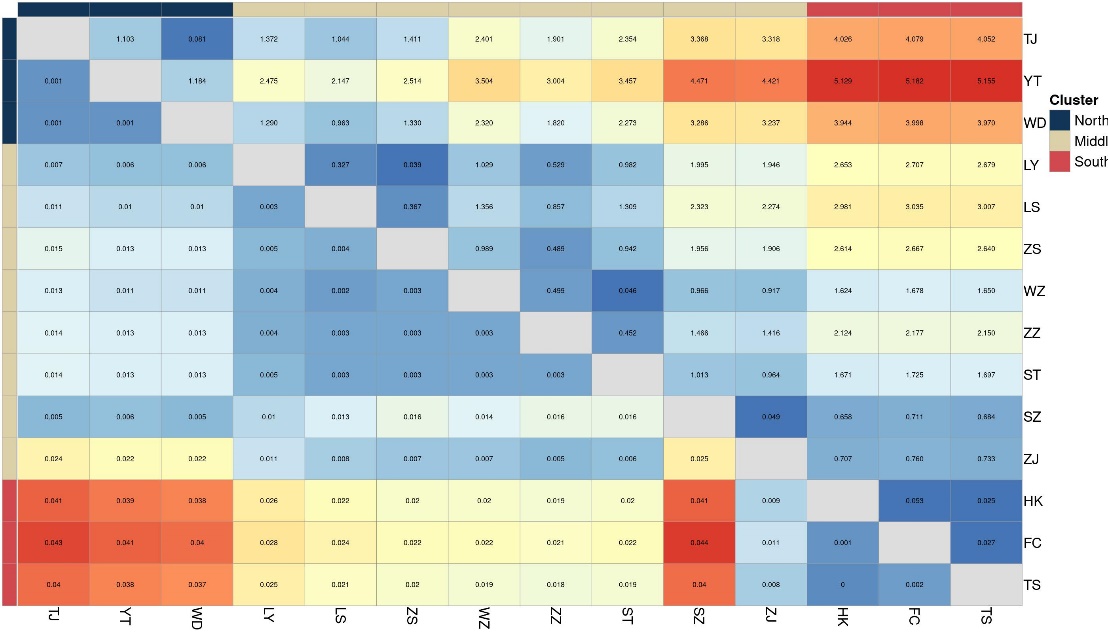


**Figure S4. A heatmap shows pairwise genetic distances and differences in maximum sea surface temperature (SST_max_) among populations.**

The pairwise genetic distances were placed in the bottom-left triangular matrix, and the pairwise SST_max_ were plotted in the top-right triangular matrix.


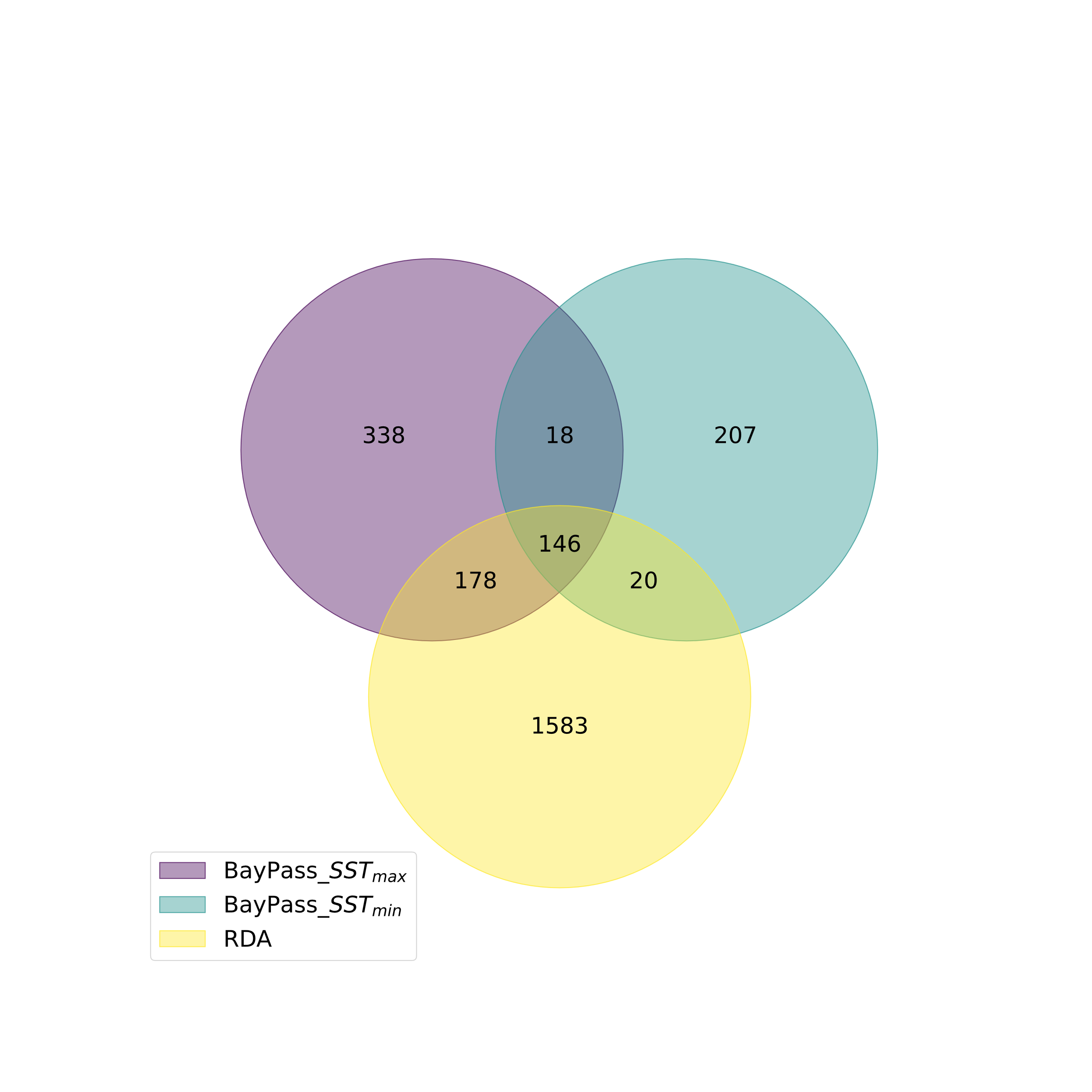


**Figure S5. A Venn plot showing overlaps between outlier SNPs detected by a Bayesian approach (BayPass) and redundancy analysis (RDA)**


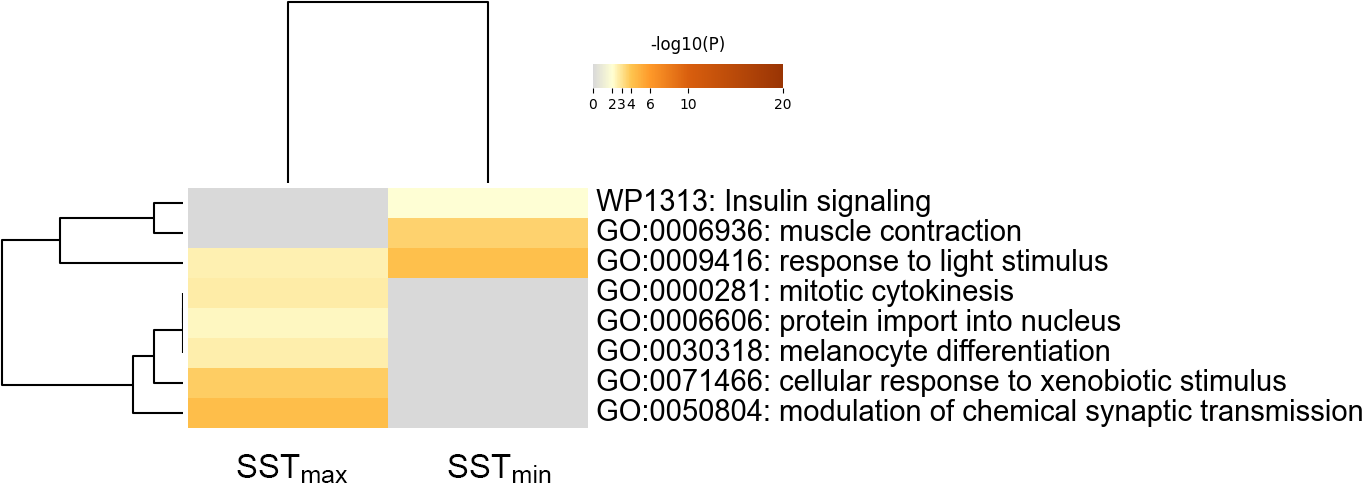


**Figure S6. A dendrogram showing enriched function clusters obtained using SST_max_- and SST_min_-associated genes**

The term with the best p-value within each cluster was selected as a representative term


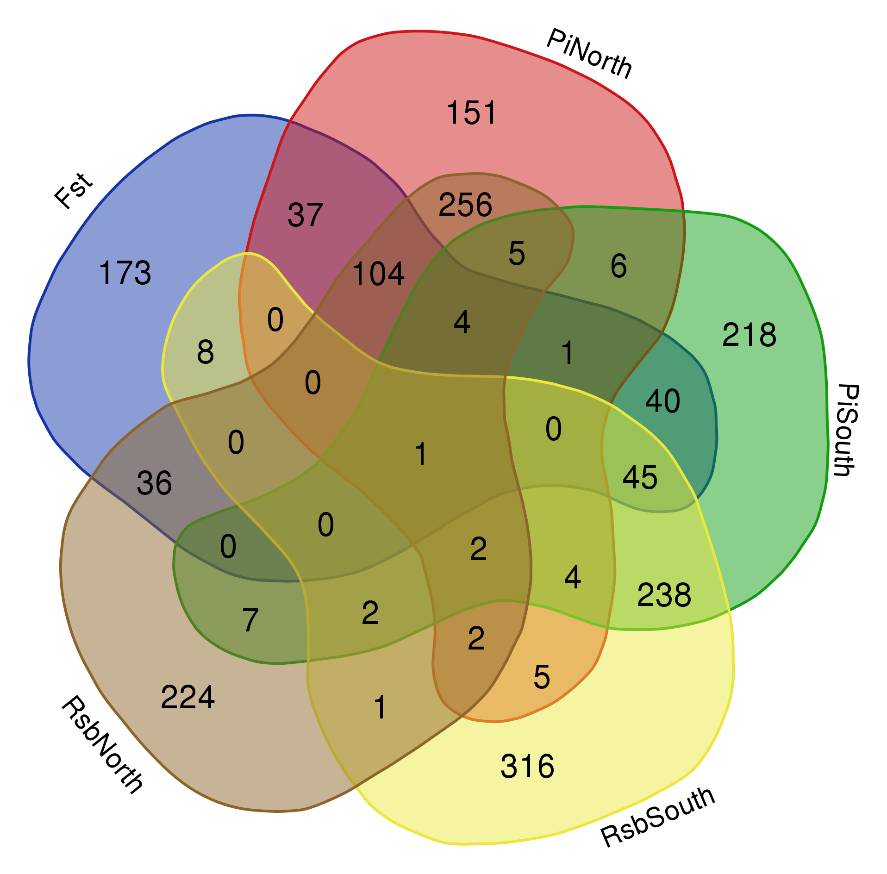


**Figure S7. A Venn plot of positively selected regions identified by different methods**


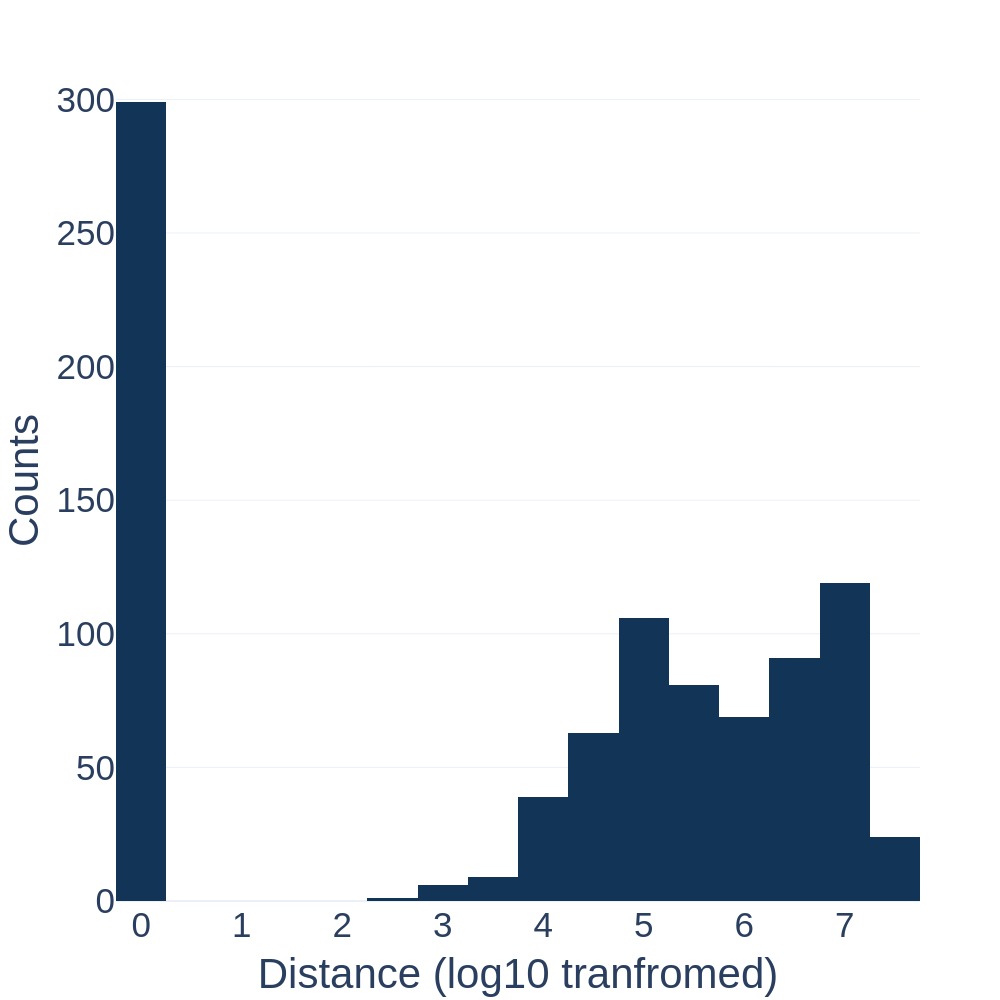


**Figure S8. Distribution of distance to the nearest PSR of temperature-associated SNP**

The distances are log-10 transformed. The distances of temperature-associated SNPs located on PSRs are calculated as zero

**
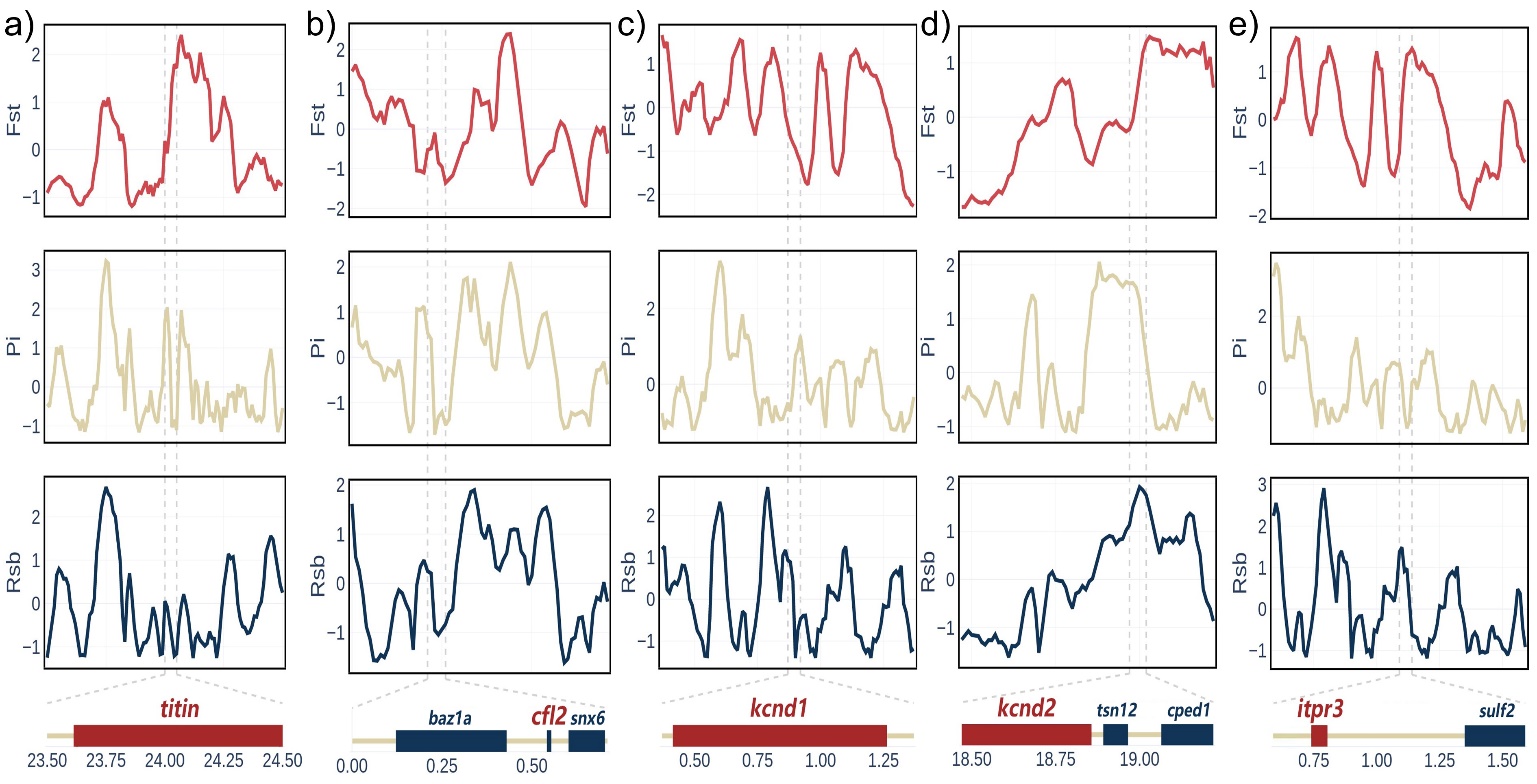
Figure S9. Adjoining selective signatures of PSGs related to fiber muscle components and muscle conduction.**

The bottom panel shows the genomic position of PSGs (the red blocks) and unselected nearby genes (the blue blocks).


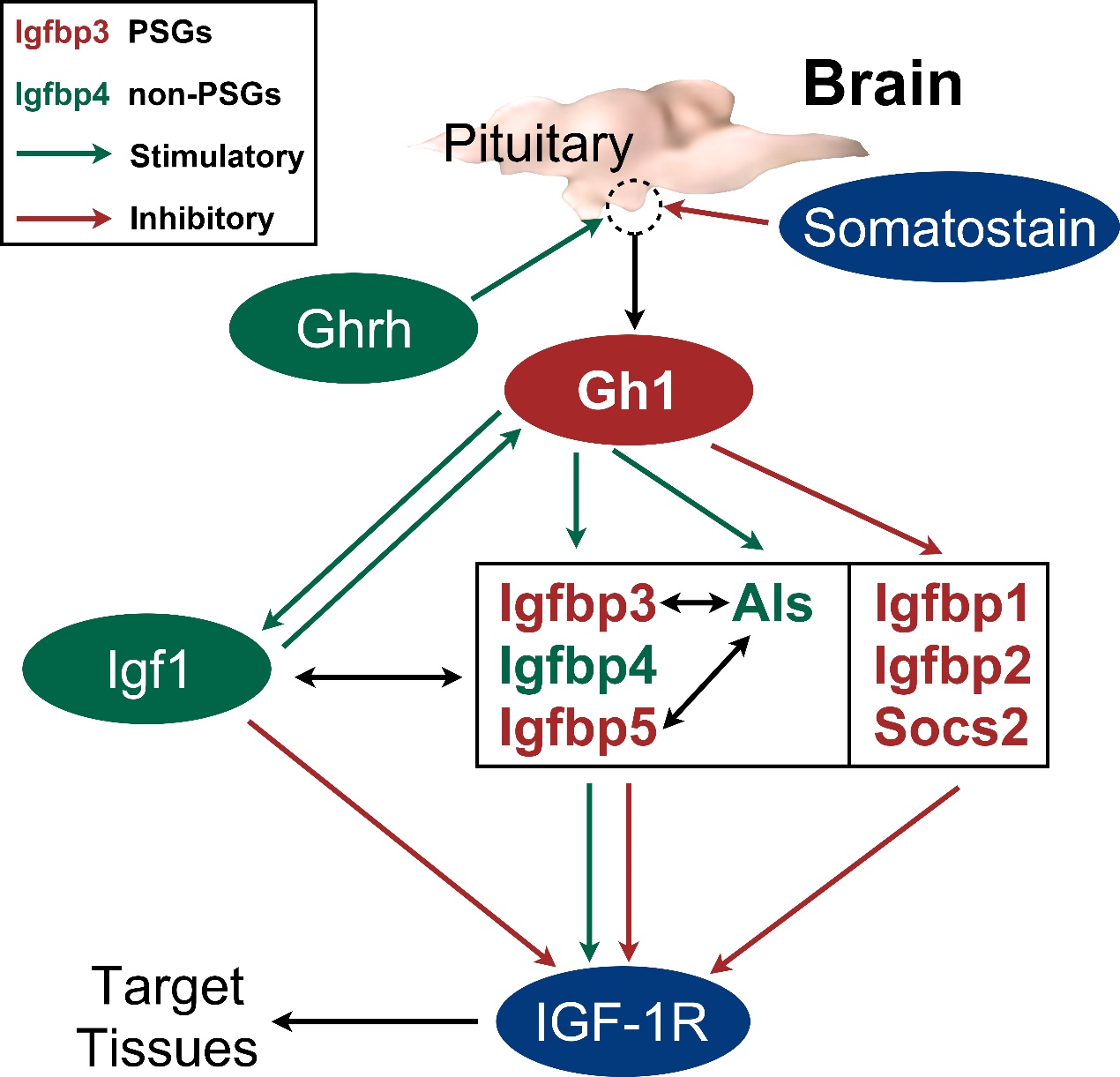


**Figure S10. A schema of GH-IGF-IGFBP axis.**

This figure was modified from Blum, Werner F. et al., 2018.

**Figure S11. Selective signatures around PSGs related to the development and regulation of the visual system
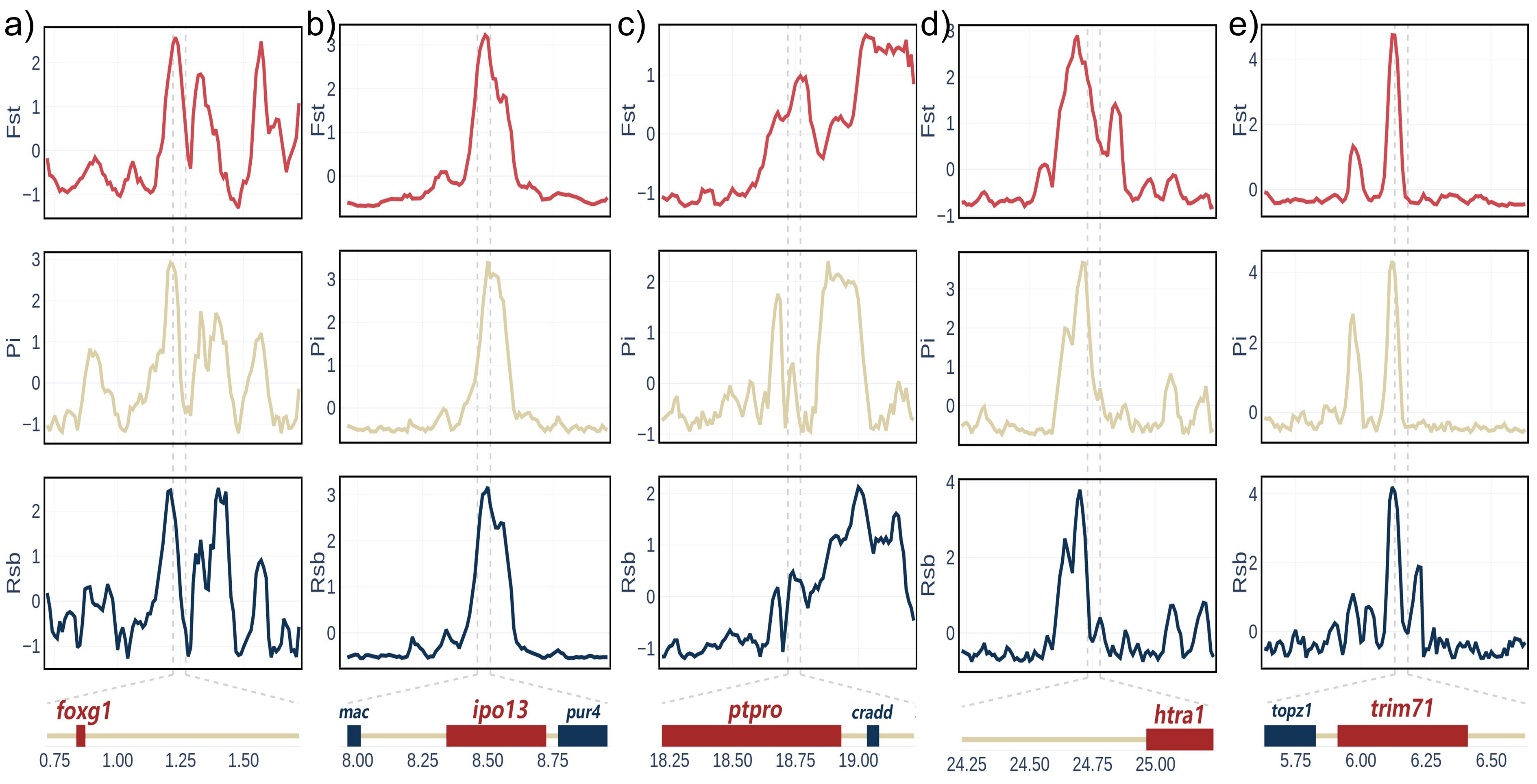
.**

The bottom panel shows the genomic position of PSGs (the red blocks) and unselected nearby genes (the blue blocks).
